# The anti-Müllerian hormone prodomain is displaced from the hormone/prodomain complex upon bivalent binding to the hormone receptor

**DOI:** 10.1016/j.jbc.2021.101429

**Published:** 2021-11-19

**Authors:** Richard L. Cate, Nathalie di Clemente, Chrystèle Racine, Nigel P. Groome, R. Blake Pepinsky, Adrian Whitty

**Affiliations:** 1 Department of Chemistry, Boston University, Boston, Massachusetts, USA; 2 INSERM, Centre de Recherche Saint Antoine (CRSA), IHU ICAN, Sorbonne Université, Paris, France; 3 School of Biological and Molecular Sciences, Oxford Brookes University, Headington, Oxford, UK; 4 Department of Biotherapeutic and Medicinal Sciences, Biogen, Cambridge, Massachusetts, USA

**Keywords:** anti-Müllerian hormone, AMHR2, TGF-β, prodomain, displacement, Ab, antibody, ActR2, activin receptor type-2, ALK1, activin receptor–like kinase 1, AMH, anti-Müllerian hormone, AMHR2, anti-Müllerian hormone receptor type-2, BMP, bone morphogenetic protein, BMPR2, bone morphogenetic protein receptor type-2, c-AMH, cleaved AMH, CHO, Chinese hamster ovary cells, DMEM, Dulbecco's modified Eagle's medium, GDF-8, growth and differentiation factor 8, GF, growth factor, HRP, horseradish peroxidase, RT, room temperature, SA, streptavidin, TGF-β, transforming growth factor-β, u-AMH, uncleaved AMH

## Abstract

Noncovalent complexes of transforming growth factor-β family growth/differentiation factors with their prodomains are classified as latent or active, depending on whether the complexes can bind their respective receptors. For the anti-Müllerian hormone (AMH), the hormone–prodomain complex is active, and the prodomain is displaced upon binding to its type II receptor, AMH receptor type-2 (AMHR2), on the cell surface. However, the mechanism by which this displacement occurs is unclear. Here, we used ELISA assays to measure the dependence of prodomain displacement on AMH concentration and analyzed results with respect to the behavior expected for reversible binding in combination with ligand-induced receptor dimerization. We found that, in solution, the prodomain has a high affinity for the growth factor (GF) (*K*_*d*_ = 0.4 pM). Binding of the AMH complex to a single AMHR2 molecule does not affect this *K*_*d*_ and does not induce prodomain displacement, indicating that the receptor binding site in the AMH complex is fully accessible to AMHR2. However, recruitment of a second AMHR2 molecule to bind the ligand bivalently leads to a 1000-fold increase in the *K*_*d*_ for the AMH complex, resulting in rapid release of the prodomain. Displacement occurs only if the AMHR2 is presented on a surface, indicating that prodomain displacement is caused by a conformational change in the GF induced by bivalent binding to AMHR2. In addition, we demonstrate that the bone morphogenetic protein 7 prodomain is displaced from the complex with its GF by a similar process, suggesting that this may represent a general mechanism for receptor-mediated prodomain displacement in this ligand family.

The transforming growth factor-β (TGF-β) superfamily of growth factors (GFs) regulate many aspects of cell growth and differentiation (reviewed in Ref. (1)). Consistent with the important roles played by these factors, a number of regulatory mechanisms have evolved that control access of TGF-β family members to their receptors (2). One important regulatory mechanism involves post-translational proteolytic processing. TGF-β family ligands are translated as dimeric precursor proteins comprising two polypeptide chains, each containing an N-terminal prodomain and a smaller C-terminal GF domain, which must undergo cleavage at dibasic or monobasic sites located between the two domains to generate the mature GF. After proteolytic processing at this site, the prodomain and GF remain noncovalently associated in a complex. In some TGF-β members, the prodomains block access of the GFs to their type I and II receptors and render the complexes latent: TGF-β (3, 4), growth and differentiation factor 8 (GDF-8) (5), and bone morphogenetic protein 2 (BMP-2) (6). In other TGF-β members, the prodomains do not block access to receptors: The noncovalent complexes of BMP-7 (7) and BMP-9 (8) have been shown to be active in biological assays.

The X-ray structure of the latent TGF-β1 complex has revealed that it is in a cross-armed conformation and that the prodomain shields the GF from recognition by receptors and alters its conformation (9). In contrast, the nonlatent BMP-9 complex was found to have an open-armed conformation, with a number of different interactions between the prodomain and the GF that may permit stepwise displacement of the prodomain by type I and II receptors (10). Subsequently, the structures of complexes of other TGF-β family members have indicated that the relationship between the conformation of the complexes and latency is more complicated. The nonlatent activin A complex has an intermediate conformation between cross-armed and open-armed, with some interactions between the prodomain and GF more similar to those in TGF-β1 (11). On the other hand, the latent GDF-8 complex is in an open-armed conformation, with latency conferred by a number of distinct features that stabilize the complex (12).

Anti-Müllerian hormone (AMH), also called Müllerian inhibiting substance, is a member of the TGF-β family involved in male and female reproductive development (13). AMH is responsible for the regression of Müllerian ducts in the male fetus; in the female fetus, the Müllerian ducts develop into the uterus, Fallopian tubes, and vagina (14). In the adult, AMH plays a role in Leydig cell differentiation and function (15, 16) and follicular development (17). AMH also has been shown to have potential roles within the nervous system (18, 19) and the hypothalamic–pituitary–gonadal axis (20, 21). AMH is synthesized as a large homodimeric precursor, which undergoes an obligatory proteolytic cleavage, generating a noncovalent complex that, similar to the BMP-7 and BMP-9, is biologically active (22, 23). Furthermore, the AMH complex can bind its type II receptor, anti-Müllerian hormone receptor type-2 (AMHR2), which leads to displacement of the prodomain (24) (Fig. 1*A*). Prodomain displacement by receptors has also been shown for BMP-7 (7) and BMP-9 (25). AMHR2 is one of the only five type II receptors that must accommodate the 30 plus TGF-β ligands, so sharing receptors is a common feature of the TGF-β family. Uniquely for the family, AMH and AMHR2 have a monogamous relationship. The crystal structure of AMH bound to AMHR2 has revealed the basis for this specificity (26).

**Figure 1:**
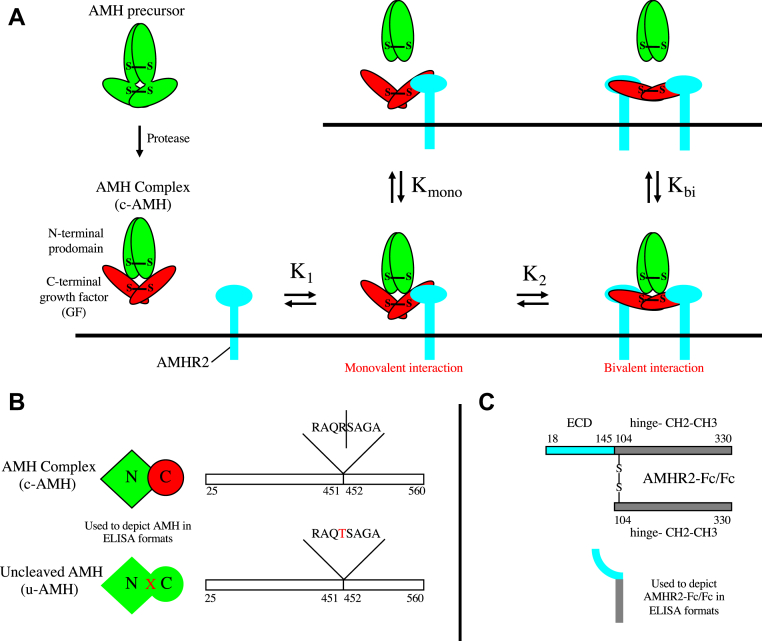
**Interaction of AMH with its type II receptor AMHR2.***A*, the AMH precursor undergoes an obligatory cleavage resulting in the noncovalent AMH complex that can bind to AMHR2, the type II receptor. After binding, the prodomain is released, but it has not been determined whether this is the result of a monovalent interaction or a bivalent interaction with AMHR2 or either interaction. In the current study, values for *K*_mono_ and *K*_bi_ have been derived showing that prodomain release occurs only after a bivalent interaction, which induces a change in the conformation of the GF. Both the GF and prodomain contain intermolecular disulfide bonds. *B*, reagents used for experiments in the current study. c-AMH was produced in Chinese hamster ovary cells as mostly precursor and converted to completely cleaved noncovalent complex by treatment with plasmin. u-AMH contains an R451T mutation at the cleavage sites between the N-terminal prodomain and the C-terminal GF domain. The numbering corresponds to amino acid residues in UniProtKB accession number P03971 (human AMH). *C*, AMHR2-Fc/Fc was generated by coexpressing a complementary DNA encoding an AMHR2-Fc fusion protein together with a complementary DNA encoding the Fc portion of IgG1 in human embryonic kidney 293 cells. The numbering corresponds to amino acid residues in UniProtKB accession numbers Q16671 (human AMHR2) and P01857 (human 1g γ-1 chain constant region). The representations of c-AMH and u-AMH (shown in *B*) and AMHR2-Fc/Fc (shown in *C*) are used to depict these molecules in ELISA formats. AMH, anti-Müllerian hormone; AMHR2, anti-Müllerian hormone receptor type-2; c-AMH, cleaved AMH; GF, growth factor; u-AMH, uncleaved AMH.

In the present study, we have investigated the nature of the interaction between the AMH complex and AMHR2 and the requirements for displacement of the prodomain. We find that binding of AMH complex to AMHR2 when it is on a surface increases the *K*_*d*_ for dissociation of the complex by a factor of over 1000, leading to rapid release of the prodomain. Displacement of the prodomain correlates with bivalent binding of the AMH complex by two molecules of AMHR2. By fitting our data to the relationships that have been derived for ligand-induced receptor dimerization (27), we are able to obtain values for the affinity of the initial monovalent binding of AMH to AMHR2 (*K*_1_) and for the subsequent recruitment of a second AMHR2 molecule to form a bivalent interaction (driven by *K*_2_) as well as for the dissociation constants for the AMH complex when bound monovalently and bivalently by the receptor (*K*_mono_ and *K*_bi_; Fig. 1*A*).

## Results

### Surface-captured AMHR2 discriminates between intact AMH complex and the mature GF and promotes prodomain displacement

We have previously shown that when the cleaved noncovalent AMH complex binds to AMHR2 on cells, or to soluble AMHR2 receptor coupled to Sepharose, the prodomain is displaced from the complex leaving the mature GF bound to the receptor (24). To quantitatively characterize these events, we developed assays to assess the binding affinity of AMH for AMHR2 under various conditions and to measure the *K*_*d*_ for dissociation of the AMH complex into separate prodomains and GF domains in solution or when bound to AMHR2. For these experiments, we used a recombinant form of AMH that is plasmin cleaved to give 100% noncovalent complex (referred to as cleaved AMH [c-AMH]; Fig. 1*B*). We employed a soluble receptor construct that contains the extracellular domain of human AMHR2 fused to the Fc portion of human immunoglobulin G. This construct is functionally monovalent as it comprises a heterodimer of one Fc-only chain disulfide bonded to one AMHR2-Fc chain (referred to as AMHR2-Fc/Fc; Fig. 1*C*) (28). This heterodimeric receptor construct was used because the AMHR2-Fc dimer we initially generated was found to contain an intermolecular disulfide bond between extracellular domains that affected AMH binding. For these assays, we also used four anti-AMH mAbs, two that bind the N-terminal prodomain (10.6 and 11F8), hereinafter referred to as mAb-N_1_ and mAb-N_2_, and two against the C-terminal GF (22A2 and F2B/12H), referred to as mAb-C_1_ and mAb-C_2_.

**Figure 2:**
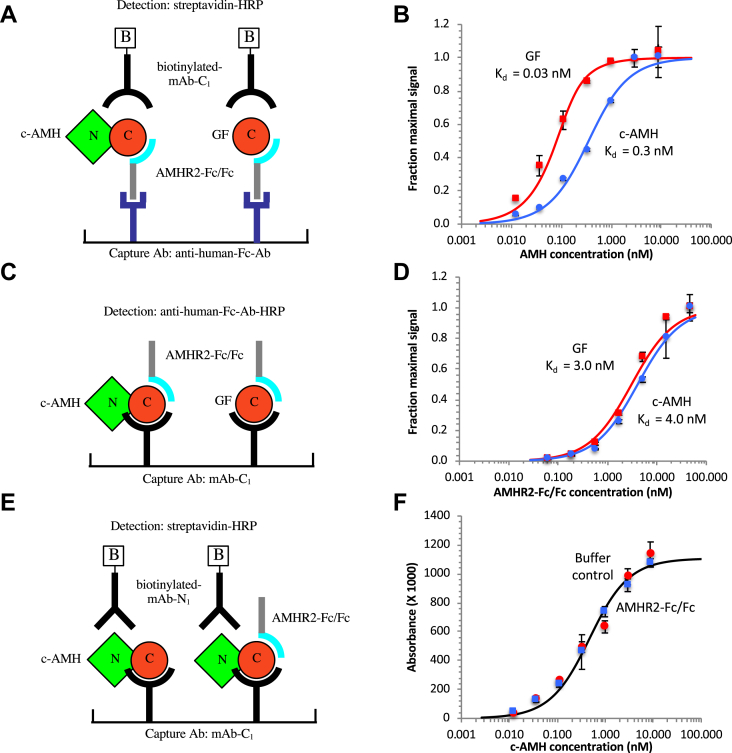
**A negative effect of the prodomain on AMH complex binding to AMHR2 is observed when AMHR2 is on a surface but not in solution.***A* and *B*, ELISA format and results for comparing binding of c-AMH or GF to AMHR2 when the receptor is on a surface. The AMHR2-Fc/Fc was captured on a goat antihuman Fc Ab and incubated with various concentrations of AMH; bound AMH was detected with biotinylated anti-C-terminal AMH mAb-C_1_ and streptavidin conjugated to horse radish peroxidase (HRP). *C* and *D*, ELISA format and results for comparing binding of c-AMH or GF to AMHR2 when the receptor is presented in solution. AMH was captured on mAb-C_1_ and incubated with various concentrations of AMHR2-Fc/Fc; bound AMHR2-Fc/Fc was detected with an antihuman-Fc Ab conjugated to HRP. *E* and *F*, ELISA format and results for assessing whether AMHR2-Fc/Fc in solution can induce displacement of the prodomain. c-AMH, after capture on mAb-C_1_, was incubated with AMHR2-Fc/Fc (33 nM) or buffer for 2 h, prior to detection with mAb-N_1_. Data in *B* and *D* were fit to the quadratic equation for a reversible association reaction. Error bars show standard deviations. Multivalent interactions are not shown in the diagrams depicting ELISA formats. Ab, antibody; AMH, anti-Müllerian hormone; AMHR2, anti-Müllerian hormone receptor type-2; c-AMH, cleaved AMH; GF, growth factor.

Two different ELISA formats shown in Figure 2, *A* and *C* were used to measure the *K*_*d*_ for the AMH–AMHR2 interaction. When the soluble receptor was captured on the assay plate surface (Fig. 2*A*), the dissociation constants of the GF and c-AMH were found to be 0.03 and 0.3 nM, respectively (Fig. 2*B*). In contrast, when the GF and c-AMH were captured on the surface and the soluble receptor was presented in solution (Fig. 2*C*), the dissociation constants were almost identical at 3 to 4 nM (Fig. 2*D*). Thus, the GF and c-AMH have higher affinities for AMHR2 when the receptor is on a surface. As demonstrated in the next section, the higher affinity is the result of an avidity effect because of a bivalent interaction of the homodimeric AMH with neighboring AMHR2 molecules on the surface. Although two separate receptor molecules can also bind AMH when the receptor is in solution, under these conditions, each interaction is independent, and no avidity enhancement of binding is expected. The results in Figure 2, *B* and *D* also show that the prodomain weakens AMH binding to the receptor, but that this effect is only seen when the receptor is presented on the solid surface. To test whether the AMH complex remained intact during the experiment shown in Figure 2, *C* and *D*, we performed a similar experiment but used mAb-N_1_ to detect whether binding of the soluble receptor caused dissociation of the AMH complex and loss of the prodomain (Fig. 2*E*). The results (Fig. 2*F*) show that no displacement of the prodomain occurred when c-AMH captured on mAb-C_1_ was incubated with a high concentration of AMHR2-Fc/Fc (33 nM) prior to detection with biotinylated mAb-N_1_.

**Figure 3:**
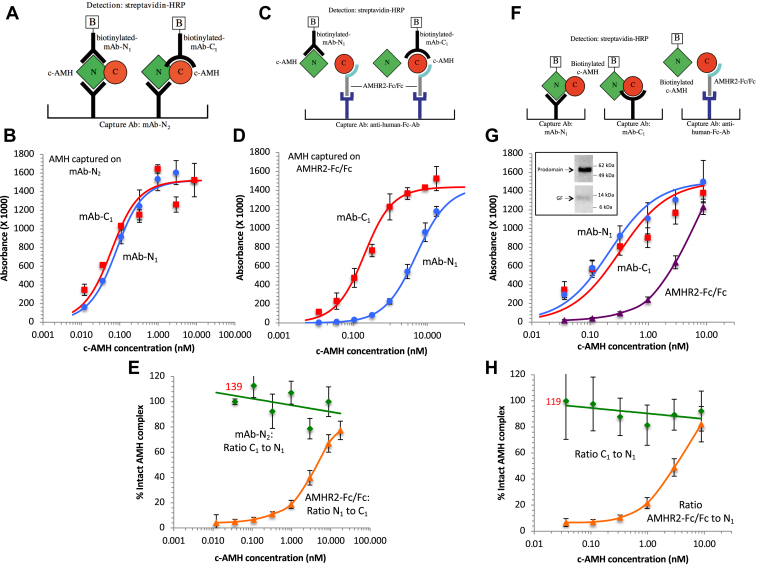
**The AMH complex undergoes dissociation after binding to AMHR2-Fc/Fc on a surface but only at low concentration.***A* and *B*, ELISA used for assessing AMH complex dissociation when bound to anti-N-terminal mAb-N_2_. Bound c-AMH was detected with either mAb-N_1_ or mAb-C_1_. Similar signals were observed with both mAbs indicating that the AMH complex has not dissociated. *C* and *D*, ELISA used for assessing AMH complex dissociation when bound to AMHR2-Fc/Fc. Less signal was obtained with mAb-N_1_ than mAb-C_1_ at low c-AMH concentrations indicating that the AMH complex has dissociated. *E*, the ratios of the signals produced by mAb-N_1_ and mAb-C_1_ in *B* and *D* are shown for a range of c-AMH concentrations and provide an estimation of the amount of intact AMH complex. *F* and *G*, ELISA used for assessing dissociation of biotinylated c-AMH when bound to mAb-N_1_, mAb-C_1_, or AMHR2-Fc/Fc. The *inset* in *G* shows a Western blot of biotinylated c-AMH showing that most of the biotin was incorporated into the N-terminal prodomain. *H*, the ratios of signals produced after binding biotinylated c-AMH to the two mAbs (C_1_/N_1_) or to AMHR2-Fc/Fc and mAb-N_1_ are shown for a range of c-AMH concentrations. The numbers in *red* in *E* and *H* show the actual ratios at these low c-AMH concentrations. Values of 100 have been used in their place so as to not skew the slopes of the trend lines. Error bars show standard deviations. AMH, anti-Müllerian hormone; AMHR2, anti-Müllerian hormone receptor type-2; c-AMH, cleaved AMH.

To further investigate the effect of receptor binding on dissociation of the AMH complex, the ELISA assays shown in Figure 3, *A* and *C* were performed. In these experiments, c-AMH was captured *via* mAb-N_2_, or *via* the soluble receptor, and the bound AMH was detected either using biotinylated mAb-N_1_ to detect the presence of the prodomain or using mAb-C_1_ to detect the GF domain. The results show that similar signals were detected with the two different biotinylated detection antibodies, mAb-N_1_ and mAb-C_1_, when the c-AMH was captured *via* mAb-N_2_ (Fig. 3*B*), indicating that the AMH complex remains intact. However, when the c-AMH was captured *via* the plate-bound receptor (Fig. 3*D*), much less signal was detected by biotinylated mAb-N_1_, relative to mAb-C_1_, indicating that the N-terminal prodomain has been displaced. The ratios of the signals obtained with mAbs C_1_ and N_1_ in Figure 3, *B* and *D* provide an estimation of how much AMH complex remains intact at each AMH concentration. Figure 3*E* shows that, while no dissociation of the complex was observed when the c-AMH was captured on the mAb, significant dissociation was seen when the c-AMH was captured on the plate-bound receptor. However, this dissociation only occurred at low concentrations of c-AMH; at higher concentrations, an increasing proportion of the AMH complex remained intact.

To confirm that the assay in Figure 3*D* was indeed detecting dissociation of the AMH complex, the experiment in Figure 3*F* was performed. In this case, the c-AMH itself was directly biotinylated on the prodomain, allowing detection with streptavidin–horseradish peroxidase (SA–HRP). This experiment exploited the adventitious observation that, upon biotinylation of c-AMH, most (>95%) of the biotin is incorporated into the N-terminal prodomain, as shown by the Western blot in the inset to Figure 3*G* (24). When the biotinylated c-AMH was captured on either mAb-N_1_ or mAb-C_1_, similar signals were obtained (Fig. 3*G*). However, when the biotinylated c-AMH was captured *via* the plate-bound receptor, much less signal was observed at low AMH concentrations, again indicative that under these conditions, the AMH complex dissociates and the biotinylated prodomain is released from the plate. The ratios of the signals measured after capturing the biotinylated c-AMH *via* the mAbs *versus* the soluble receptor provide an estimate of how much AMH complex is still intact at each AMH concentration (Fig. 3*H*). Consistent with the results obtained previously using the antibody (Ab) detection, the c-AMH remained intact when captured on the plate *via* the antibodies, but considerable dissociation of c-AMH was observed when captured *via* the plate-bound receptor, although again only at low AMH concentrations.

**Figure 4:**
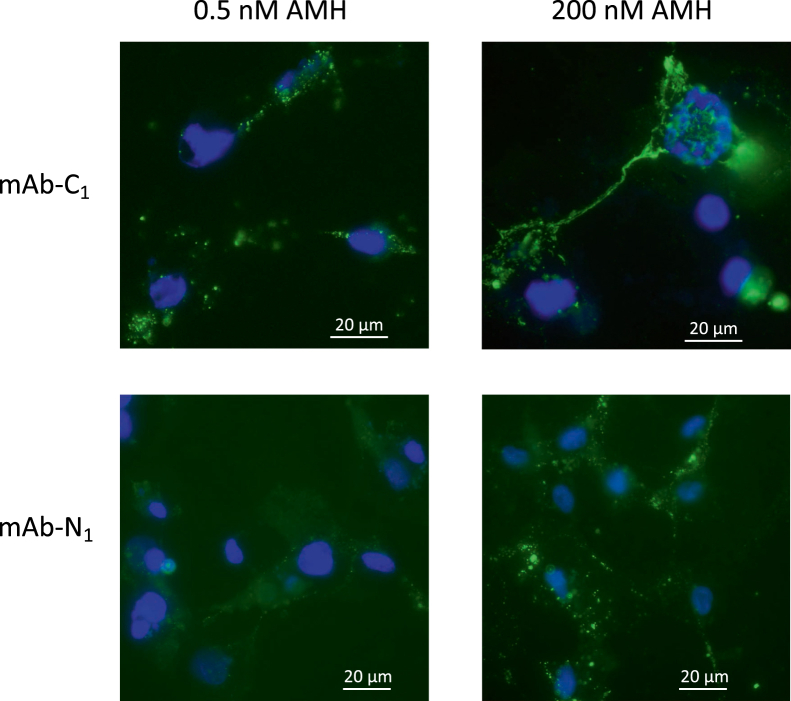
**The prodomain can be detected after binding of c-AMH to AMHR2 on COS cells at high concentration but not after binding at low concentration.** COS cells were transfected with AMHR2 and incubated with c-AMH at 0.5 or 200 nM. AMH bound to cells was detected with either mAb-C_1_ or mAb-N_1_ as described in the Experimental procedures section. AMHR2, anti-Müllerian hormone receptor type-2; c-AMH, cleaved AMH.

In previous experiments with COS cells expressing AMHR2, we showed that AMH complex bound at low concentration was detectable with mAb-C_1_, but not with mAb-N_1_, consistent with the prodomain being displaced (24). This result is confirmed in Figure 4, when c-AMH was bound at a low concentration (0.5 nM) to COS cells expressing AMHR2. However, when c-AMH was bound at a high concentration (200 nM), both mAbs could now detect the bound AMH, indicating that the prodomain was still present. Thus, the phenomena observed in the ELISAs of prodomain displacement occurring at low AMH concentration but not high concentration could also be recapitulated on cells.

### Dissociation of the AMH complex correlates with bivalent binding to AMHR2

**Figure 5:**
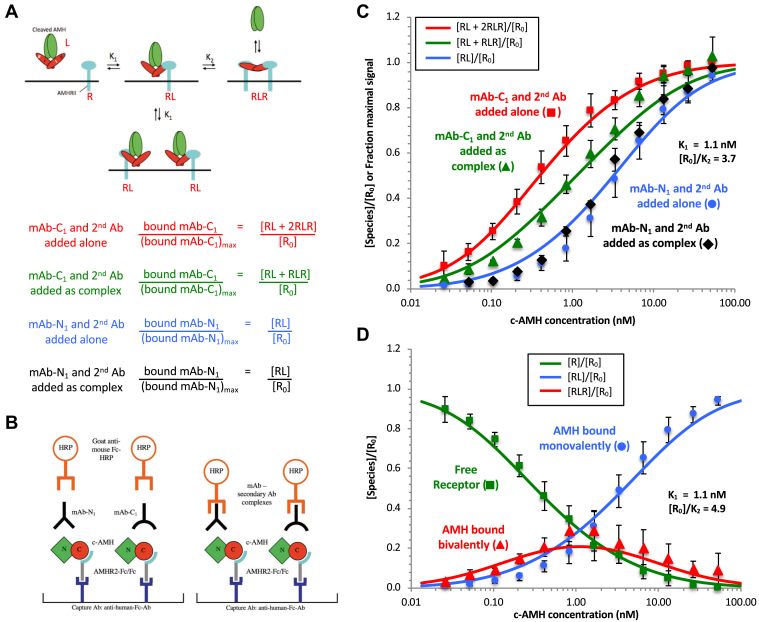
**Dissociation of the AMH complex correlates with bivalent binding to AMHR2.***A*, a mechanism for the receptor-induced dimerization of AMHR2 by c-AMH; monomeric and bivalent interactions are labeled RL or RLR, respectively. The relationships between mAb-N_1_ or mAb-C_1_ and RL and RLR are shown below the diagram. *B*, schematic diagrams showing the two experimental conditions for testing whether bivalent binding of AMH by AMHR2 induces prodomain displacement. On the *left*, the mAbs and secondary Ab are added individually, and on the *right*, they are added as a complex. *C*, data from Table 1 obtained with mAb-N_1_ and mAb-C_1_ under the two experimental conditions shown in *B* fit to the equations of Perelson and DeLisi. *D*, dependence of [R]/[R_0_], [RL]/[R_0_], and [RLR]/[R_0_] on AMH complex concentration. In *C* and *D*, *K*_1_ and [R_0_]/*K*_2_ values were determined by simultaneously fitting all data to the predicted curves and minimizing the RMSE. Error bars show standard deviations. Ab, antibody; AMH, anti-Müllerian hormone; AMHR2, anti-Müllerian hormone receptor type-2; c-AMH, cleaved AMH.

**Figure 6:**
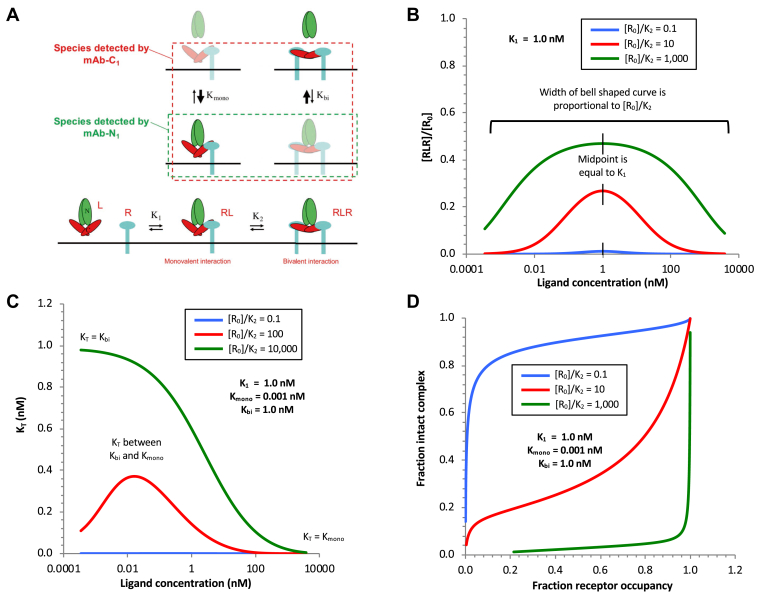
**The effect of the [R**_**0**_**]/*K***_**2**_**ratio on the level of ligand bound bivalently and on complex dissociation.***A*, the two equilibrium states for the AMH complex interacting with AMHR2 monovalently or bivalently are shown in relation to the species detected by mAb-N_1_ and mAb-C_1_. The species detected by mAb-N_1_ (shown in the *green dashed rectangle*) are related to [RL], whereas the species detected by mAb-C_1_ (shown in the *red dashed rectangle*) are related to [RL + RLR] or [RL + 2RLR], depending on experimental conditions as described in the text. The effect of varying the [R_0_]/*K*_2_ ratio is shown for [RLR]/[R_0_] (*B*), *K*_T_ (*C*), and the relationship between complex dissociation and receptor occupancy (*D*). Curves were generated using the equations of Perelson and DeLisi alone (*B*) or in combination with the quadratic equation for a reversible dissociation/association reaction (*C* and *D*) and the indicated values of *K*_1_, [R_0_]/*K*_2_, *K*_mono_, and *K*_bi_ as described in the Experimental procedures section. AMH, anti-Müllerian hormone; AMHR2, anti-Müllerian hormone receptor type-2.

The simplest hypothesis that accounts for the concentration dependence of the dissociation of the c-AMH complex is that it can bind to the plate-captured receptor in two different states, one that is prone to dissociation and one that is not. Specifically, we hypothesized that, at low AMH concentrations, this homodimeric protein can bind to two neighboring molecules of the monovalent AMHR2 on the plate, whereas at high AMH concentrations, mass action requires that each AMHR2 molecule on the assay plate will bind a separate AMH dimer, interacting with only one of the polypeptide subunits of the GF domain. This concentration-dependent shift from bivalent to monovalent binding is a well-known feature of systems in which a bivalent ligand binds to a surface-presented monovalent receptor (27, 29). If this hypothesis is correct, the observation that prodomain displacement is seen predominantly at low AMH implies that a bivalent interaction with the receptor is required for dissociation of the AMH complex. To test this hypothesis, we fitted our ELISA data using the equations that have been previously derived for ligand-induced receptor dimerization (27, 29) (see the Experimental procedures section). The proposed mechanism for AMH-induced dimerization of AMHR2 is shown in Figure 5*A*, with monovalent and bivalent interactions labeled RL or RLR, respectively; the equations of Perelson and DeLisi allow the calculation of [RL] and [RLR] over a range of c-AMH concentrations at specific values of *K*_1_, the affinity of the initial monovalent binding of ligand to the first receptor molecule, and *K*_2_, the affinity of the subsequent recruitment of the second receptor molecule into the bivalent complex. As Figure 6*A* shows, the form of the AMH complex detected by mAb-C_1_ can be related to [RL + RLR], as this Ab will detect all forms of bound AMH whether or not the prodomain remains associated. Meanwhile, if our hypothesis that binding the receptor bivalently promotes dissociation of the prodomain is correct, the species detected by mAb-N_1_ can be related to [RL], as the monovalently bound RL will experience little loss of the prodomain, whereas there will remain little or no prodomain to be detected in RLR (Fig. 6*A*).

To generate suitable data for comparison to this mechanistic model, we developed ELISA methods to detect bound AMH using nonbiotinylated mAbs and a secondary antimouse-Fc Ab conjugated to HRP, added either together as a preformed complex or individually in two steps (Fig. 5*B*). We found that, when mAb-C_1_ is added as a preformed complex, only one detection Ab complex binds per bound AMH molecule, whether the AMH is bound monovalently (RL) or bivalently (RLR). Thus, monitoring bound AMH in this manner gives a signal that is proportional to the sum of [RL + RLR]. In contrast, when mAb-C_1_ is added alone, two molecules of this Ab can bind per AMH dimer, one to each polypeptide, whether the AMH is present as RL or RLR. However, while the subsequently added HRP-conjugated antimouse-Fc Ab can detect both mAb-C_1_ molecules bound to the RLR complex, only one of the bound mAb-C_1_ molecules is detected in the case of the RL complex. The reasons for this two-to-one bias in detecting RLR *versus* RL, when the antibodies are added separately, are explained in Figs. S1 and S2. Thus, the signal obtained by this method is proportional to [RL + 2RLR]. A third way of detecting bound AMH was by using the prodomain-targeted Ab mAb-N_1_ together with the antimouse-Fc Ab. According to our hypothesis, this combination will predominantly recognize RL but not RLR because in the latter case the prodomain has mostly dissociated from the complex. Thus, the signal given in such assays should be approximately proportional to [RL] (Fig. S3). The data obtained using mAb-N_1_ were unaffected by whether the mAb-N_1_ and the antimouse-Fc Ab were added sequentially or as a preincubated complex, consistent with the expectation that this method detects only a single species. Overall, therefore, we can use mAb-C_1_/antimouse-Fc added together to measure [RL + RLR], which corresponds to the total amount of bound AMH, mAb-C_1_, and antimouse-Fc added sequentially to measure [RL + 2RLR], which corresponds to the level of receptor occupancy, and mAb-N_1_/antimouse-Fc to measure primarily [RL], which corresponds to c-AMH bound monovalently to receptor (summarized in Fig. 5*A*).

We performed experiments similar to the one in Figure 3*D*, except using the nonbiotinylated mAbs detected using antimouse Fc, as described previously. The data (Table 1) were globally fitted to the equations of Perelson and DeLisi, giving values for *K*_1_ of 1.1 nM and [R_0_]/*K*_2_ of 3.7 (Fig. 5*C*). Note that, because in our experiments, the receptor is captured on a solid surface, it is not appropriate to treat *K*_2_ as a molar equilibrium constant. Instead, [R_0_]/*K*_2_ is a dimensionless number that represents the ratio of receptor involved in bivalent *versus* monovalent complexes at low receptor occupancy (*i.e.*, when bivalent binding is most favored because there is no shortage of unoccupied receptor available to form bivalent complexes). Importantly, almost identical results were obtained with two different prodomain- and GF-specific mAbs, which bind to different epitopes in AMH (Fig. S4*A*). Also, an ELISA that detected RL by means of biotinylated c-AMH, performed as in Figure 3*F*, gave results similar to those obtained using mAb-N_1_ (Fig. S4*B*).

**Table 1:** Data from ELISA with mAb-N_1_ and mAb-C_1_ shown in Figure 5*C*

[c-AMH] (nM)	Determined experimentally			Calculated from values in columns A, B, or C			
	mAb-C_1_/(mAb-C_1_)_max_ (mAb-C_1_ & second Ab added alone)	mAb-C_1_/(mAb-C_1_)_max_ (mAb-C_1_ & second Ab added as complex)	mAb-N_1_/(mAb-N_1_)_max_				
	[RL+2RLR]/[R_0_] Fraction-receptor occupancy	[RL+RLR]/[R_0_] Fraction-maximal AMH binding	[NC_T_]/[R_0_] Also estimate of [RL]/[R_0_]∗	[N_T_]/[R_0_] or [C_T_]/[R_0_] Also estimate of [RLR]/[R_0_]∗	[R]/[R_0_] Fraction-free receptor	*K*_T_ (pM) (at [R_0_] = 100 pM)^#^	[NC_T_]/[RL + RLR] Fraction-intact AMH complex
52.83	1.000 ± 0.000	1.026 ± 0.087	0.941 ± 0.022	0.086 ± 0.090	0.000 ± 0.000	0.8 ± 0.0	0.917 ± 0.080
26.41	0.989 ± 0.018	0.965 ± 0.008	0.871 ± 0.041	0.094 ± 0.041	0.011 ± 0.018	1.0 ± 0.6	0.903 ± 0.043
13.21	0.951 ± 0.038	0.940 ± 0.042	0.794 ± 0.064	0.145 ± 0.077	0.049 ± 0.038	2.7 ± 2.0	0.845 ± 0.078
6.60	0.917 ± 0.036	0.854 ± 0.048	0.654 ± 0.081	0.199 ± 0.094	0.083 ± 0.036	6.1 ± 4.1	0.767 ± 0.104
3.30	0.834 ± 0.042	0.707 ± 0.048	0.487 ± 0.082	0.220 ± 0.095	0.166 ± 0.042	10.0 ± 6.3	0.688 ± 0.125
1.65	0.789 ± 0.073	0.596 ± 0.060	0.311 ± 0.079	0.285 ± 0.099	0.211 ± 0.073	26.2 ± 14.5	0.521 ± 0.142
0.83	0.654 ± 0.068	0.463 ± 0.041	0.178 ± 0.055	0.284 ± 0.069	0.346 ± 0.068	45.3 ± 20.8	0.386 ± 0.124
0.41	0.538 ± 0.072	0.315 ± 0.037	0.109 ± 0.028	0.206 ± 0.047	0.462 ± 0.072	38.7 ± 15.8	0.347 ± 0.097
0.21	0.386 ± 0.054	0.203 ± 0.017	0.056 ± 0.018	0.147 ± 0.025	0.614 ± 0.054	36.6 ± 15.7	0.276 ± 0.093
0.10	0.254 ± 0.036	0.125 ± 0.003	0.034 ± 0.009	0.091 ± 0.009	0.746 ± 0.036	24.3 ± 7.2	0.271 ± 0.071
0.05	0.163 ± 0.037	0.088 ± 0.023	0.021 ± 0.009	0.067 ± 0.025	0.837 ± 0.037	22.1 ± 15.0	0.234 ± 0.119
0.03	0.103 ± 0.065	0.043 ± 0.006	0.013 ± 0.002	0.031 ± 0.006	0.897 ± 0.065	7.5 ± 2.6	0.290 ± 0.067
	A	B	C	D	E	F	G
Calculations	--	--	--	B - C	1 - A	(D^2^/C)R_0_	C/B
N	5	4	6				

Calculations were performed as indicated at the bottom of the table and in Fig. S3*A*. Errors are standard deviations. N refers to the number of experiments; each experiment contained 2 to 4 replicates. ∗Values of [RL]/[R_0_] and [RLR]/[R_0_] are estimates based on assumption that [RL] = [bound mAb-N_1_] when *K*_mono_ is small and *K*_bi_ is large (see Fig. S3*B*). ^#^*K*_T_ = [N_T_][C_T_]/[NC_T_]. Experimental and theoretical values for *K*_T_ were calculated using a value of [R_0_] = 0.1 nM, determined from experimentation. Values of [R_0_] twice as high (0.2 nM) or half as high (0.05 nM), give similar results when fitting the data to the model (Fig. S5), indicating that having an exact value of [R_0_] is not essential for this analysis.

Values for [R]/[R_0_] and [RLR]/[R_0_] were calculated from the experimental datasets as described in Table 1. Figure 5*D* shows how the concentrations of RL, RLR, and free R vary as a function of AMH concentration. Global fitting of these three datasets to the equations of Perelson and DeLisi gave values of *K*_1_ = 1.1 nM and [R_0_]/*K*_2_ of 4.9, which are in close agreement with the best fit values obtained from Figure 5*C*. As expected, the relationship of [RLR]/[R_0_] to AMH concentration is a bell-shaped curve, with the value of *K*_1_—the dissociation constant for the monovalent interaction of the AMH complex with AMHR2—corresponding to its midpoint. This value of *K*_1_ = 1.1 nM agrees well with the value of 3 to 4 nM obtained for the monovalent AMH/AMHR2 interaction measured in solution (Fig. 2*D*). The best-fit value obtained for the ratio [R_0_]/*K*_2_ was 4.9 (Fig. 5*D*). Figure 6*B* shows how the RLR curves are predicted to look at different values of [R_0_]/*K*_2_. Taken overall, the close fit of the data to the predicted curves in Figure 5*D* strongly supports the notion that bivalent interaction of the AMH complex with the receptor results in displacement of the prodomain, whereas little or no dissociation of the AMH complex occurs when bound monovalently.

### Bivalent binding to AMHR2 increases the *K*_*d*_ for dissociation of the AMH complex by at least 1000-fold

**Figure 7:**
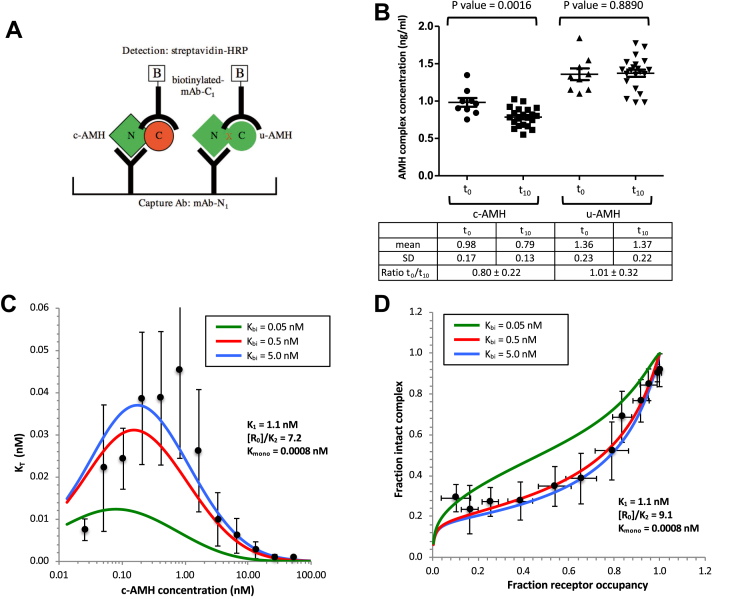
**Bivalent binding of AMHR2 to the AMH complex increases the *K***_***d***_**of the complex by a factor of 1000.***A*, ELISA used to measure intact AMH complex. *B*, c-AMH and u-AMH were diluted in PBS containing 1% BSA and 1% goat serum to 1 ng/ml (8.8 pM), and the level of intact AMH complex was assessed after 10 days (*t*_10_; N = 21); samples diluted immediately before the ELISA are labeled *t*_0_ (N = 9). *C*, concentration dependence of the apparent dissociation constant (*K*_T_) determined for the AMH complex bound to AMHR2. *K*_T_ values are from Table 1. *D*, relationship between complex dissociation and receptor occupancy. Data for intact AMH complex [NC_T_]/[RL + RLR] and receptor occupancy [RL + 2RLR]/[R_0_] are from Table 1. In *C* and *D*, the curves were generated using the quadratic equation for a reversible dissociation/association reaction in combination with the equations of Perelson and DeLisi. *K*_1_ and *K*_bi_ were fixed at 1.1 and 5 nM, respectively, and values for *K*_mono_ and [R_0_]/*K*_2_ were determined by minimizing the RMSE as described in the Experimental procedures section. The effect of varying *K*_bi_ is shown. Error bars show standard deviations. AMH, anti-Müllerian hormone; c-AMH, cleaved AMH; u-AMH, uncleaved AMH.

To learn more about how receptor binding affects the interaction between the AMH prodomain and GF domain, we first measured the *K*_*d*_ for dissociation of the AMH complex in solution. To do this, c-AMH was diluted to a concentration of 1 ng/ml (8.8 pM) and incubated for 10 days at room temperature (RT). As a control, a preparation of recombinant AMH that contains a mutation at the cleavage site and thus is not susceptible to proteolytic cleavage and therefore cannot dissociate (uncleaved AMH [u-AMH], Fig. 1*B*) was treated in a similar way. At the end of the incubation, the AMH levels were determined using the ELISA shown in Figure 7*A*, which detects only intact complex. After incubation for 10 days, the mean level of c-AMH measured was 80% of the mean level measured in samples diluted immediately before the assay (Fig. 7*B*); an unpaired *t* test indicated that this difference was significant at a confidence level of *p* = 0.0016. In contrast, no significant reduction in mean AMH concentration was observed in the u-AMH samples after 10 days compared with samples diluted immediately before the assay. The fraction of intact c-AMH complex observed after the 10 days of incubation was used to calculate an estimated *K*_*d*_ for the complex, giving a value of 0.4 pM.

To measure the dissociation constants of the AMH complex when bound monovalently (*K*_mono_) or bivalently (*K*_bi_) to AMHR2, we modeled our experimental data using the quadratic equation for a reversible dissociation/association reaction in combination with the equations of Perelson and DeLisi (Fig. 7*C*). In our experimental design, it is not possible to directly measure *K*_mono_ or *K*_bi_. However, it is possible to measure the overall amount of receptor-bound AMH, which corresponds exactly to the species detected by mAb-C_1_, [RL + RLR], as well as the total amount of bound AMH that has remained intact, which corresponds exactly to the species detected by mAb-N_1_ (see Fig. S3*A*). The signal detected by mAb-N_1_, in principle, could include intact AMH bound either monovalently (NC_mono_) or bivalently (NC_bi_). Thus, mAb-N_1_ detects the sum of these species, [NC_T_], where [NC_T_] = [NC_mono_] + [NC_bi_]. By subtracting [NC_T_] from the total AMH bound, [RL + RLR], we can also quantify the amount of bound AMH that no longer has an associated prodomain, which we termed [C_T_] (=[C_mono_] + [C_bi_]). This treatment does not require the approximation, made in the preliminary analysis described previously, that mAb-N_1_ detects only RL. The results of these calculations are shown in Table 1.

The total distribution of bound AMH between prodomain-dissociated and intact forms can be described by the following equations:

$$K_{\text{T}}=\left[{\text{N}}_{\text{T}}\right]\left[{\text{C}}_{\text{T}}\right]/\left[{\text{NC}}_{\text{T}}\right]$$

$$K_{\text{T}}=\left[{\text{N}}_{\text{bi}}{+\text{N}}_{\text{mono}}\right]\left[{\text{C}}_{\text{bi}}{+\text{C}}_{\text{mono}}\right]/\left[{\text{NC}}_{\text{bi}}{+\text{NC}}_{\text{mono}}\right]$$

The quantity *K*_T_ is not a dissociation constant but corresponds to the weighted average of *K*_mono_ and *K*_bi_ that pertains under particular experimental conditions. Values for *K*_T_, calculated from the experimental data measured at each AMH concentration tested, are given in Table 1 and Figure 7*C*. The results show that *K*_T_ has low values at both low and high AMH concentrations, indicating that there is less or little dissociation of the AMH complex at these extremes of concentration but passes through a maximum value indicating that substantial release of prodomain occurs at intermediate [AMH].

To investigate whether this observed bell-shaped relationship between *K*_T_ and AMH concentration is expected for our proposed mechanism from Figure 5*A*, we modeled how the value of *K*_T_ would vary as a function of [c-AMH] for this mechanism, using the equations of Perelson and DeLisi to model the distribution of bound AMH between monovalent and bivalent receptor engagement and the quadratic equation for reversible binding to determine the extent of dissociation of prodomain from the plate-bound AMH. The parameters that determine the behavior of this model are the values of *K*_1_, [R_0_]/*K*_2_, *K*_mono_, and *K*_bi_, as shown in Figure 6*A*. Specifically, *K*_1_ determines the overall extent of receptor occupancy that will be seen at a given AMH concentration, [R_0_]/*K*_2_ determines the distribution of bound AMH between monovalent (RL) and bivalent (RLR) complexes, and *K*_mono_ and *K*_bi_ determine the extent to which the monovalently bound (RL) or bivalently bound (RLR) AMH complexes will dissociate to release the prodomain into solution. If [R_0_]/*K*_2_ is a large number (Fig. 6*C*), then at low [AMH], when there is plenty of unbound receptor available, a substantial fraction of the bound ligand will be recruited into RLR complexes. Consequently, the *K*_T_ value at low ligand concentrations will approximate *K*_bi_. On the other hand, at high AMH concentrations that force the system into a predominantly monomerically bound state, *K*_T_ will approximate *K*_mono_. Moderate AMH concentrations, at which there is a mix of bivalently and monovalently bound AMH, will lead to *K*_T_ values that lie between *K*_bi_ and *K*_mono_. In contrast, if [R_0_]/*K*_2_ is small (Fig. 6*C*), such that there is no significant recruitment of bound AMH into RLR complexes at any AMH concentration, then *K*_T_ will equal *K*_mono_ at all concentrations of ligand. The model reveals that if [R_0_]/*K*_2_ has an intermediate value, such that there are significant amounts of both monovalently bound and bivalently bound AMH even at low AMH concentrations where bivalent binding is most favored, the *K*_T_ value will vary from *K*_mono_ at high ligand concentrations, through a maximum at moderate ligand concentrations, to a value at the lowest ligand concentrations that is small but still intermediate between *K*_bi_ and *K*_mono_.

Figure 7*C* and Table 1 show that the experimentally determined *K*_T_ values range from a high of around 45 pM at low c-AMH concentration to a low of 0.8 to 1.0 pM at high c-AMH concentration. The latter range of values provides a measure of *K*_mono_ and is very close to the *K*_*d*_ value determined for the AMH complex in solution. This finding indicates that monovalent binding of c-AMH to a single AMHR2 molecule does not significantly promote c-AMH dissociation and release of the prodomain. Values for *K*_bi_ of ≥5 nM give the best fit between the model and the experimental data, with values for *K*_bi_ less than 0.5 nM being clearly inconsistent with the data. Thus, engagement of a second molecule of AMHR2 to form a bivalent complex decreases the affinity of the prodomain for the GF by at least 1000-fold, compared with unbound c-AMH, from ∼0.4 pM to ≥0.5 nM.

Figure 7*D* represents another test of the hypothesis that a bivalent interaction induces dissociation of the AMH complex. Curves were generated by plotting the fraction of intact AMH complex, given by [NC_T_]/[RL + RLR], against the fraction receptor occupancy, [RL + 2RLR]/[R_0_]. Figure 6*D* shows how the model predicts this relationship will differ for low, high, and intermediate values of [R_0_]/*K*_2_. At low values of [R_0_]/*K*_2_, the affinity for recruiting a second receptor molecule is low, and there is essentially no bivalent binding at any ligand concentration. Thus, there is little or no release of prodomain, except at very low levels of receptor occupancy, where the ligand concentration is below even the very low *K*_*d*_ for dissociation of monovalently bound complex. At high values of [R_0_]/*K*_2_, under which conditions bivalent binding is strongly favored except at extremely high ligand concentrations, loss of prodomain is almost complete at all levels of receptor occupancy except the very highest because only at extremely high ligand concentrations will the equilibrium favor RL. Finally, at intermediate values of [R_0_]/*K*_2_, such as we see experimentally in this study, the model predicts a gradual increase in the fraction of ligand that remains intact from low to high receptor occupancy, as the proportion of RL to RLR gradually increases. Figure 7*D* shows the experimental values for the fraction of bound c-AMH that is intact at different AMH concentrations from Table 1 (column G) plotted against the level of receptor occupancy determined from the signal measured using mAb-C_1_ (column A), together with the best fit to the model. Fitting to the model gives values for *K*_mono_ and *K*_bi_ of 1 pM and ≥0.5 nM, respectively, consistent with the results obtained from Figure 7*C*.

### Displacement of the prodomain from the BMP-7 complex

Because the activation mechanism we observed for AMH differs, in key respects, from the activation mechanisms reported for other TGF-β family members (7, 25), we decided to directly compare prodomain displacement from the AMH complex to prodomain displacement from another TGF-β family ligand. We chose BMP-7 for this comparison because, for this ligand too, it has been shown that prodomain release is induced by binding to its type II receptor (bone morphogenetic protein receptor type-2 [BMPR2]). Unlike for AMH, however, for BMP-7, it was reported that the soluble receptor can achieve this activation (7). Experiments on BMP-7 that exactly parallel those shown in Figures 5 and 7 were not possible because the mAb against the BMP-7 prodomain that at one time was commercially available has only low affinity, whereas the mAb against the GF is neutralizing and thus may block interaction with the type II receptor. We therefore prepared BMP-7 that was biotinylated on just the prodomain or just the GF domain for our measurements. These materials were prepared by biotinylating the BMP-7 complex, separating the biotinylated protein into prodomain and GF fractions, and then combining each biotinylated component into a complex with its nonbiotinylated counterpart.

**Figure 8:**
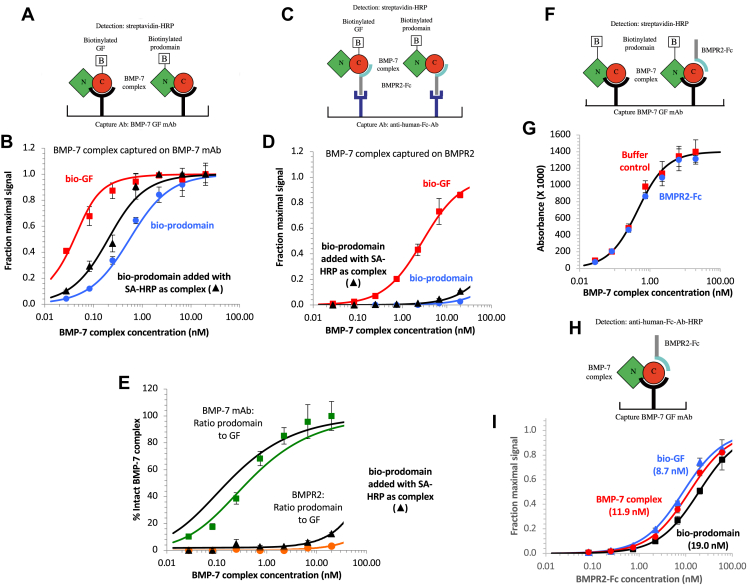
**Displacement of the prodomain from the BMP-7 complex.***A* and *B*, ELISA format and results for assessing BMP-7 complex dissociation when bound to BMP-7 GF mAb. BMP-7 complexes containing either biotinylated prodomain or GF were captured on the BMP-7 mAb, and the amount of bound prodomain or GF was assessed using streptavidin (SA)–HRP. *C* and *D*, ELISA format and results for assessing BMP-7 complex dissociation when bound to BMPR2-Fc. In *B* and *D*, assays were also performed in which the biotinylated prodomain preparation was added as a preformed complex with SA–HRP. *E*, the ratios of the signals produced by biotinylated prodomain and GF in *B* and *D* are shown for a range of BMP-7 concentrations and provide an estimation of the amount of intact BMP-7 complex. The concentration dependence of the dissociation of the BMP-7 complex captured on the BMP-7 mAb indicates that the *K*_*d*_ of the complex is around 0.1 to 0.2 nM. The data for the biotinylated prodomain protein added as a complex with SA–HRP are not shown, only the curve fitted to the data. Much lower levels of intact complex were detected after capture on BMPR2-Fc, indicating that the prodomain has been displaced by the receptor. *F* and *G*, ELISA format and results for assessing whether BMPR2-Fc in solution can induce displacement of the prodomain. BMP-7 complex containing biotinylated prodomain was captured on the BMP-7 mAb and incubated with BMPR2-Fc (60 nM) or buffer for 2 h, prior to detection with SA–HRP. No prodomain displacement was observed relative to the buffer control. *H* and *I*, ELISA format and results for assessing binding of BMPR2 to the two biotinylated proteins and the unbiotinylated BMP-7 complex. Similar *K*_*d*_ values were obtained for the three proteins. Error bars show standard deviations. BMP-7, bone morphogenetic protein 7; BMPR2, bone morphogenetic protein receptor type-2; GF, growth factor; HRP, horseradish peroxidase.

To probe the mechanism of prodomain displacement, the prodomain-biotinylated or GF-biotinylated BMP-7 complexes were captured on an ELISA plate either using an anti-BMP-7 GF mAb or *via* BMPR2-Fc, shown schematically in Figure 8, *A* and *C*. When captured on the anti-BMP-7 mAb, reduced signals were observed for the prodomain relative to the GF domain at low concentrations of BMP-7 (Fig. 8*B*), indicating that a significant amount of complex dissociation has occurred. Assays were also performed in which the biotinylated prodomain preparation was added as a preformed complex with SA-HRP in order to eliminate the incubation step with SA-HRP after binding of the biotinylated prodomain protein to the mAb. As expected, less dissociation was observed (Fig. 8*B*). When captured on BMPR2-Fc, almost no signal was detected with the biotinylated prodomain preparation, when added individually or as a complex with SA-HRP (Fig. 8*D*). The ratios of the signals obtained with the two biotinylated complexes in Figure 8, *B* and *D* provide an estimate of how much BMP-7 complex remains intact at each concentration (Fig. 8*E*). In contrast to the AMH complex, considerable dissociation was observed when the BMP-7 complex was captured on the mAb, an indication that the *K*_*d*_ of the BMP-7 complex is higher than that of the AMH complex; modeling indicates a value of *K*_*d*_ = 0.1 to 0.2 nM. An even higher level of complex dissociation was observed when the BMP-7 complex was captured on the plate-bound receptor, indicating that interaction with receptor further weakens the complex, consistent with the prior literature (7). Displacement of the prodomain at low concentrations of complex is most likely caused by bivalent interactions with BMPR2. The decrease in displacement at the highest complex concentrations, observed more prominently when the biotinylated prodomain preparation was added as a preformed complex with SA-HRP, suggests that a monovalent interaction with BMPR2 may not cause dissociation of the BMP-7 complex. However, to prove this by going to higher BMP-7 complex concentrations was not possible because of the limited availability of the biotinylated prodomain complex.

To assess whether an interaction between the complex and receptor in solution can induce displacement, biotinylated prodomain complex was captured using an anti-BMP-7 GF mAb and then incubated with or without a high concentration of BMPR2-Fc for 2 h (Fig. 8*F*). As shown in Figure 8*G*, no prodomain displacement was observed with soluble receptor. The experiment in Figure 8, *H* and *I* shows that BMPR2-Fc, added in solution, binds to the biotinylated prodomain complex with a *K*_*d*_ value similar to that of the biotinylated GF complex and unbiotinylated BMP-7 complex. Together, the experiments in Figure 8, *G* and *I* indicate that BMPR2 can bind to the BMP-7 complex without inducing further displacement of the prodomain. This finding suggests that, as was the case with AMH, prodomain displacement from the BMP-7 complex requires a bivalent interaction with surface-bound BMPR2. However, we cannot rule out the possibility that, in Figure 8*G*, the bound BMP-7 can bind soluble BMPR2-Fc only monovalently, if receptor binding to the other monomer in the homodimeric GF is blocked by the interaction with the anti-BMP-7 GF mAb. Thus, it remains possible that two BMPR2 molecules binding to the BMP-7 complex in solution might induce prodomain displacement. These results therefore indicate that displacement of the BMP-7 prodomain occurs by a mechanism similar to that seen with AMH, involving binding of type II receptor molecules to both GF polypeptides within the complex, but leave open the question of whether surface presentation of the receptor is absolutely required in the case of BMP-7.

## Discussion

The regulatory mechanisms that control access of TGF-β family members to their receptors are of central importance to understanding the physiology of TGF-β signaling (2). An important mechanism for regulating the maturation of some TGF-β ligands is the association of the proteolytically cleaved prodomain with the GF. In some cases, this noncovalently bound prodomain prevents interaction with receptors and renders the complex latent. However, for other family members, the cleaved ligand complexes are not latent and can interact with their receptors. The latter is true for the cleaved AMH complex, which can bind its type II receptor in an interaction that then induces displacement of the prodomain (24). In this report, we have investigated the effect of AMHR2 on the prodomain–GF interaction in the AMH complex and the effect of the prodomain on the AMH–AMHR2 interaction. We show that binding of the AMH complex to a single AMHR2 molecule has no detectable effect on the stability of the prodomain–GF complex, but recruitment of a second AMHR2 to form a bivalent complex with ligand increases the *K*_*d*_ for the ligand complex by a factor of over 1000, resulting in rapid dissociation of the prodomain. Importantly, this effect is only observed when AMHR2 is presented on a surface; binding of soluble AMHR2 to c-AMH did not lead to prodomain displacement.

It is usually difficult to discriminate monovalent and bivalent interactions between receptors and their ligands or to obtain precise measurements for the affinity of the receptor dimerization step. In this study, we exploited the fact that, for bivalent ligands binding to an identical monovalent receptor on a surface, the relative abundances of monovalently bound and bivalently bound ligand vary in a predictable manner, with the abundance of bivalently bound ligand showing a characteristic bell-shaped dose response (27). Using this feature, and antibodies that allowed us to detect whether the bound AMH molecules did or did not contain prodomain at each condition, we were able to determine the affinity constants for all steps in the reaction. In particular, we were able to determine the value for the affinity for the initial monovalent binding of c-AMH to the first equivalent of AMHR2, *K*_1_, as well as the value for the quantity [R_0_]/*K*_2_ that reflects the affinity for recruitment of a second AMHR2 to form the bivalently bound RLR complex. [R_0_]/*K*_2_ is a dimensionless quantity that corresponds to the preponderance of bivalently bound receptor compared with monovalently bound receptor that exists at conditions of low receptor occupancy where all nearby receptor molecules are available for recruitment into a bivalent complex. The value we obtained for [R_0_]/*K*_2_ = ∼6 indicates that, under the conditions of our experiments, at low levels of receptor occupancy, approximately six times as many receptor molecules are engaged in bivalent complexes (RLR) compared with monovalent complexes (RL). The numerical value of [R_0_]/*K*_2_ will depend not only on the affinity of the final step in bivalent binding (*K*_2_) but also on the density of AMHR2 present on the surface ([R_0_]). In the current work, the significance of this value is mainly that it was large enough for us to observe a substantial degree of bivalent binding, which allowed us to characterize how this bivalent receptor engagement affected the affinity of prodomain binding. However, in previous work, we have shown that binding of c-AMH to AMHR2 on cells similarly results in displacement of the prodomain (24), establishing that receptor-induced prodomain displacement also occurs in a cellular context. In the current work, characterizing these events *in vitro* has allowed us to establish the detailed mechanism by which this process occurs.

The bell-shaped curve observed for bivalently bound AMH in Figure 5*D* (RLR) spans about four logs of ligand concentration. Other homodimeric receptors are known to exhibit bell-shaped dose responses. For example, hGH has an effective dose range of 4 to 6 logs, whereas erythropoietin and prolactin display broad effective dose ranges of six logs and greater than six logs, respectively (30). These very large effective dose ranges indicate that the affinity for the initial and monovalently bound ligand complex on the cell surface to recruit a second receptor molecule to form a bivalent and functional complex is very high. Consequently, these bivalent complexes form at very low ligand concentration and require extremely high concentrations of ligand to outcompete the bivalent binding to force the system into a state where each receptor is occupied by a separate ligand molecule in a nonfunctional monovalent complex (30). The similarity between the bell-shaped curve for the bivalently bound AMH complex (RLR) and the bell-shaped effective dose ranges of these other cytokines suggests that AMHR2 has also evolved a high interaction affinity for recruitment of a second molecule of AMHR2 after the initial monovalent binding of AMH to the first AMHR2 molecule. The affinity enhancement that results from bivalent binding may have particular importance in extragonadal targets of AMH action such as the nervous system (19, 31), where AMH levels may be low. It should be noted that determination of an exact value of [R_0_]/*K*_2_ for the reaction as it occurs on cells expressing a given level of AMHR2 would require experimentation on those cells (30).

The similarity between the *K*_*d*_ for dissociation of the AMH complex in solution (0.4 pM) determined directly *versus K*_mono_ (0.8–1.0 pM) determined by fitting our data to the equations of Perelson and DeLisi indicates that monovalent binding to AMHR2 does not substantially affect the dissociation affinity of the AMH complex. The low *K*_*d*_ for the AMH complex in solution indicates that most of the complex is intact in biological fluids, an unsurprising result given that the mature GF is relatively unstable in the absence of the prodomain (23). AMH levels are present in the range of 25 to 50 pM in human female serum (age 5–30 years; (32)), a concentration at which 84 to 87% of AMH would exist as the intact complex. Even higher AMH levels (>250 pM) are present in male serum prior to puberty (33), corresponding to a level of intact complex of >94%. On the other hand, the dissociation constants for the BMP-4, BMP-5, BMP-10, and GDF-8 complexes have been reported to be in the range of 4 to 13 nM (34), whereas *K*_*d*_ for the activin A complex is 5 nM (11). Most of these factors show bioactivity and are present in biological fluids at concentrations below 1 nM, such that a significant level of dissociation would be expected. For example, activin A levels range from a value of 10 pM in female serum during the menstrual cycle to as high as 1 nM during the third trimester of gestation (35). Even at the higher level of 1 nM, at a *K*_*d*_ of 5 nM, only ∼15% of the complex would remain intact at equilibrium absent some other mechanism for preventing dissociation. The low *K*_*d*_ of the AMH complex is most likely because of an avidity effect since, unlike many members of the TGF-β family, the AMH prodomain has been shown to exist as a disulfide-linked dimer (22, 23).

The *K*_*d*_ for the release of the prodomain from the AMH complex bound bivalently to AMHR2, *K*_bi_, was found to be ≥500 pM, indicating that bivalent engagement of the AMH complex by AMHR2 increases the dissociation constant by a factor of ≥1000. An important property of allosteric interactions is that they are reciprocal in nature (36, 37, 38). Therefore, since AMHR2 can bind to the GF in the AMH complex and displace the prodomain, the prodomain must have a reciprocal effect on the binding affinity of the GF to AMHR2. In previous experiments, we failed to find an effect of the prodomain on binding affinity for AMHR2; similar affinities were observed for the mature domain and the AMH complex binding to AMHR2 (24). However, in these earlier experiments, the AMH was presented on a surface, and the receptor protein was presented in solution. As shown in Figure 2, when the receptor is presented on a surface, the C-terminal GF does indeed have a higher affinity for AMHR2 than does the AMH complex. Thus, the prodomain and AMHR2 have reciprocal allosteric effects on the GF when the receptor is presented on a surface.

The fact that only surface-presented AMHR2 induces release of the prodomain shows that it is not occupancy of the second AMHR2 binding site on c-AMH, *per se*, that drives the allosteric effect. Rather, the proximity or mutual orientation of the AMHR2 molecules within the bivalent RLR complex must also be important. We therefore propose a model in which the c-AMH molecule in its solution conformation is not optimally organized to bind simultaneously to two surface-presented AMHR2 molecules, and that some conformational rearrangement is required for bivalent binding to occur. The >1000-fold effect of this conformational change on prodomain binding suggests that, in c-AMH that is unbound or bound only monovalently, this change in the structure of the AMH complex is energetically unfavorable by at least 16.5 kJ/mol (4 kcal/mol). It is the binding energy generated by the interaction of AMH with the second AMHR2 molecule that pays for this unfavorable conformational change. Thus, the value of [R_0_]/*K*_2_ that is observed experimentally, though relatively high, in fact underestimates the true strength of interaction of monovalently bound c-AMH with a second AMHR2 presented nearby on a surface. This is because a substantial amount of this binding energy is not expressed as affinity but rather is used to force the ligand into a significantly less stable conformation that promotes prodomain displacement to generate the fully mature GF (39).

**Figure 9:**
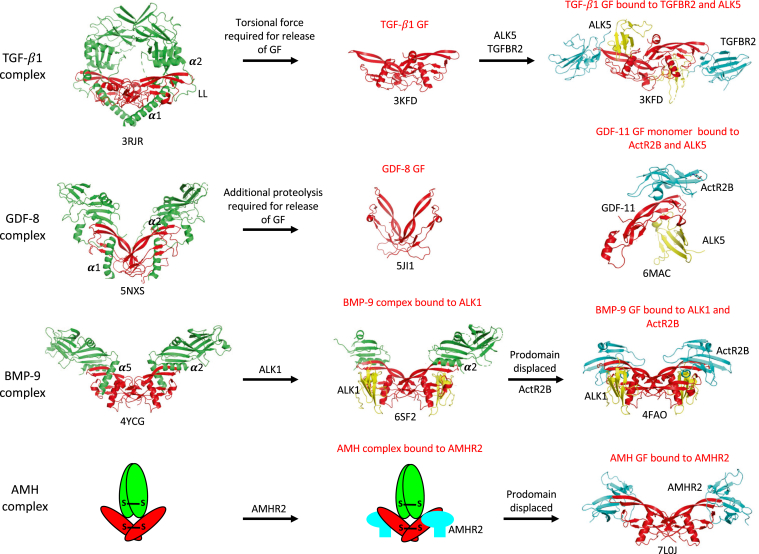
**Summary of how various TGF-β family ligands access their receptors.** The latent TGF-β1 and GDF-8 complexes require either torsional force or additional proteolysis to liberate their growth factors (GFs) and allow access to their receptors. The GF of GDF-11, which is closely related to GDF-8, is shown bound to its receptors. The nonlatent BMP-9 complex can bind to its type I receptor, ALK1, without inducing prodomain displacement. Prodomains are shown in *green*, GFs in *red*, type I receptor ECDs in *yellow*, and type II receptor ECDs in *cyan*. The Protein Data Bank file names are shown beneath each structure. The α1-, α2-, and α5-helices and latency lasso (LL) in the prodomains are labeled; these elements are located in close proximity to receptor binding sites on the GFs. ALK1, activin receptor–like kinase 1; BMP-9, bone morphogenetic protein 9; ECD, extracellular domain; GDF-8, growth and differentiation factor 8; TGF-β, transforming growth factor beta.

X-ray crystal structures of complexes of TGF-β ligands (TGF-β1 (9), BMP-9 (10), activin A (11), and GDF-8 (12)) have revealed how the prodomains of these hormones interact with their GFs, showing that certain elements within the prodomains come into close proximity with the binding sites for both the type I and type II receptors (reviewed in Ref. (2)). In these systems, the so-called “latency lasso,” α2-helix, and β1-strand of the prodomain form close associations with the type II receptor binding site (in TGF-β1, this interaction is predominantly with the latency lasso alone), whereas the α1-helix or α5-helix forms a close association with the type I receptor binding site. However, the extent to which these associations actually block access to receptors is an open question. Latent complexes clearly cannot bind their receptors, but in these cases, the GFs are confined by their prodomains in a more restricted state, which requires either torsional force for the TGF-β1 complex (9) or proteolysis for the GDF-8 complex (12) to liberate the GF and allow interaction with receptors. On the other hand, nonlatent complexes can bind their receptors without the prodomain dissociating. The BMP-9 complex can form a ternary complex with the type I receptor, activin receptor–like kinase 1 (ALK1) (40), despite the association of the α5-helix with the type I receptor binding site (10). In fact, the X-ray structure of ALK1 with the BMP-9 complex shows that ALK1 displaces the α5-helix from the type I receptor binding site but does not affect the interaction of the α2-helix and β1-strand with the type II binding site on the GF (40). During proteolytic activation of the latent GDF-11 complex, a fragment of the prodomain containing the α1-helix and α2-helix remains associated with the GF without affecting binding to the type II receptor ActRIIA or bioactivity and is not displaced after binding of ActRIIA (41). Figure 9 provides a summary of how the TGF-β1, GDF-8, and BMP-9 complexes access their receptors.

A sequence alignment of AMH with other TGF-β ligands (10) indicates that the β1-strand and α2-helix are conserved in the AMH prodomain, so it is likely that these elements also form an association with the type II receptor binding site on the GF. AMH is missing a feature that may stabilize the α2-helix interaction with the type II binding site in the BMP-9 complex: Arg-128 at the center of a π-cation cage formed by Trp-179, Phe-230, and Tyr-65 (10). In AMH, the residue homologous to Arg-128 is replaced by leucine, and two residues corresponding to those within the cage are replaced with nonaromatic amino acids. Because the *K*_*d*_ for the AMH complex is around 10^−12^ M, and given that the association rate constant between two proteins is unlikely to exceed 10^7^ M^−1^s^−1^ (42), the dissociation rate constant is likely to be no greater than 10^−5^ s^−1^, and consequently, the half-life for the dissociation of the complex (in the presence of AMHR2) will be in the range of ≥20 h. This time range is not consistent with the kinetics of the AMH complex binding to AMHR2, which is on the order of minutes. Thus, binding to the receptor must precede prodomain displacement. Furthermore, we have shown here that binding of AMHR2 to c-AMH does not require prior displacement of the prodomain. Figure 2*F* indicates that AMHR2, AMH prodomain, and AMH GF can form a stable ternary complex at a very high concentration of AMHR2. For this to be possible, it appears that the association between the β1-strand and the α2-helix of the prodomain and the type II binding site of the GF, if it occurs in the AMH complex, must be in equilibrium with a different conformational state that allows access of AMHR2 to its binding site on the GF. This picture of events is supported by our observation that *K*_mono_ is essentially identical to the *K*_*d*_ of the unbound complex, indicating that the binding of one AMHR2 molecule to the complex does not lead to any significant destabilization of the complex.

There are a number of differences in the mechanism of AMH prodomain displacement by AMHR2 and the mechanisms of prodomain displacement used by other TGF-β family members. For example, dissociation of the BMP-9 complex can be induced by the type I receptor ALK1, type II receptors BMPR2, ActR2A, ActR2B, and the coreceptor endoglin (25). In addition, antibodies to either the prodomain or GF domain can induce dissociation of the BMP-9 complex. The finding that the BMP-9 complex can form a ternary complex with the type I receptor ALK1 (40) appears to be in conflict with the finding that ALK1 can displace the prodomain (25), but it should be noted that the former experiment was done in solution, whereas the latter experiment was performed with an ALK1-Fc fusion protein on a biosensor chip where bivalent engagement by the receptor is possible. Another family member for which prodomain displacement can be induced by binding to its receptor is BMP-7. It was previously reported that BMPR2-Fc can induce BMP-7 complex dissociation when added as a soluble reagent, based on the sedimentation properties of the BMP-7 complex when combined with BMPR2-Fc in sucrose gradients (7). This result differs from our findings with AMH, for which prodomain displacement requires that AMHR2 is presented on a surface. In our own experiments on BMP-7, we found that binding to surface-bound receptor induces prodomain displacement for BMP-7 similarly to AMH, though the intrinsic affinity of the BMP-7 complex is >250-fold weaker. We did not observe enhanced prodomain displacement when we used the BMPR2 as a soluble reagent, suggesting that surface presentation of the receptor is required for BMP-7 as it is for AMH. If so, this finding would suggest a similar allosteric mechanism, requiring a constrained geometry between two receptor molecules, could be operative for both these ligands. However, we were not able to formally exclude the possibility that BMPR2 binding can induce prodomain displacement when in solution if both receptor binding sites in the GF domain of BMP-7 are simultaneously occupied.

In the AMH system, dissociation of the AMH complex strictly requires bivalent interaction with AMHR2 on a surface, which we conclude is the result of an allosteric effect. In considering the potential biological utility of this mode of receptor activation for AMH signaling, we note that the large reduction in prodomain affinity for the GF after bivalent binding to AMHR2 is likely to render prodomain displacement essentially irreversible, thus driving the receptor activation process forward. Such a mechanism in addition ensures that prodomain release will occur only when the cleaved ligand binds to cells that express AMHR2 at sufficient levels to favor formation of a c-AMH–(AMHR2)_2_ ternary complex (43). Ligand binding to cells that express only low levels of AMHR2 will form few such ternary complexes, with most ligand molecules instead simply dissociating from the cell surface as the intact GF–prodomain complex. Thus, we speculate that this mechanism may serve to ensure that the mature GF is generated only at the site of action, with a greater selectivity for cells having high receptor expression than would be expected for alternative prodomain release mechanisms and under conditions where receptor engagement by AMH is predicted to become kinetically irreversible.

## Experimental procedures

### Reagents

AMH proteins were previously described (23, 24). AMH produced in Chinese hamster ovary (CHO) cells expressing a wildtype AMH gene and purified from the culture medium is referred to as secreted-AMH. It contains about 95% AMH precursor and 5% cleaved AMH. c-AMH was prepared by plasmin digestion of secreted AMH and has been assessed as 100% cleaved by SDS-PAGE. u-AMH was produced in CHO cells expressing an AMH complementary DNA with a mutation (R451T) at the monobasic cleavage site and has been assessed as 0% cleaved by SDS-PAGE. c-AMH was biotinylated as previously described (24). The AMH GF was prepared as previously described (23). The AMHR2-Fc/Fc fusion protein was produced in humane embryonic kidney 293E cells as previously described (28) and was purified from conditioned medium using Protein A Sepharose and size-exclusion chromatography. The mAbs used in this study have been previously described: mAb-C_1_ (22A2; (44)), mAb-C_2_ (F2B/12H; (45)), mAb-N_1_ (10.6; (23)), and mAb-N_2_ (11F8; (44)).

### ELISAs

Conditions for ELISAs with mAb-N_1_ and mAb-C_1_ coated on plates have been previously described (24). Conditions for an ELISA with AMHR2-Fc/Fc have also been previously described (28, 33). Briefly, ELISA plates (Nunc Maxisorp) were coated with a goat antihuman Fc Ab (10 μg/ml; Jackson ImmunoResearch; catalog no.: 109-005-098) overnight at 4 °C in 50 mM sodium bicarbonate, pH 9.6. After washing with water and blocking with 1% bovine serum albumin (Sigma–Aldrich; A-7906) and 1% goat serum (Invitrogen; 16210064) in PBS, AMHR2-Fc/Fc was added at a concentration of 0.75 μg/ml and incubated for 1 h. After washing with PBS, AMH samples were serially diluted down the plate by a factor of 2 or 3 and incubated for 2 to 4 h. After washing with PBS/0.05% Tween-20, biotinylated mAb-N_1_ or mAb-C_1_ (0.5 μg/ml) was added and incubated for 1 h, followed by a 1 h incubation with SA–HRP (1;3000; Jackson ImmunoResearch). After washing, 3,3′,5,5′ tetramethylbenzidine was added to each well, reactions were quenched by the addition of 2 M sulfuric acid, and absorbances were read at 450 nm. When nonbiotinylated mAbs were used for detection, the subsequent incubation was with goat antimouse-Fc conjugated to HRP (1:3000; Jackson ImmunoResearch). Preformed complexes between mAbs and the goat antimouse-Fc conjugated to HRP were generated by mixing the mAbs and secondary Ab at a 1 to 2 ratio, with mAbs at a concentration of 50 nM. After incubation at 90 min at RT, the complexes were diluted 1:20 and added to the plate. When biotinylated-c-AMH was used, it was directly detected with SA–HRP. c-AMH or u-AMH captured on mAbs-N_1_ and N_2_ was detected as described previously with biotinylated mAb-N_1_ or mAb-C_1_. All antibodies were coated as described previously for the goat antihuman Fc Ab.

### Binding of AMH to AMHR2 on COS cells

COS cells were transfected with the human AMHR2 complementary DNA as previously described (24). After washing with Dulbecco's modified Eagle's medium (DMEM), cells were incubated with c-AMH (0.5 or 200 nM) in DMEM/1% fetal bovine serum for 4 h at 37 °C, then washed with DMEM, and incubated with mAb-N_1_ or mAb-C_1_ (3 μg/ml) in DMEM/1% fetal bovine serum for 2 h at 37 °C. After rinsing with PBS, cells were incubated 1 h with a goat antimouse immunoglobulin G Ab conjugated to Alexa 488 in DMEM, washed with PBS, and fixed 5 min in methanol/acetone (v/v). After hydration, slides were mounted in Vectashield containing 4′,6-diamidino-2-phenylindole (Vector Laboratories), and cells were examined with an Olympus IX83 microscope.

### Biotinylation of BMP-7

Full-length human BMP-7 was expressed in CHO cells and purified from the medium using sequential chromatography steps on Zn-chelating Sepharose and SP-Sepharose as previously described (41). The isolated BMP-7 was >98% pure and eluted as a homogeneous peak when analyzed by size-exclusion chromatography. About ∼90% was cleaved at the junction of the prodomain and mature domain to form the active mature domain/prodomain-soluble BMP-7 complex, and the remainder was not fully processed. Two different methods were used to biotinylate the soluble BMP-7. In method 1, the soluble BMP-7 was reduced with 5 mM sodium metaperiodate and biotinylated through the sugars using 3 mM Easy-link hydrazide PEG4 Biotin (Thermo Fisher Scientific). In method 2, soluble BMP-7 was biotinylated through amines using 0.3 mM EZ-Link NHS-PEG4-Biotin. The three preparations with and without biotin were loaded onto Zn-chelating Sepharose columns at 4 mg BMP-7/ml resin. Mature BMP-7 was selectively eluted from the columns with 6 M urea, and the prodomain was then eluted with 50 mM imidazole. By SDS-PAGE, the mature BMP-7 was >95% pure and the prodomain was 90% pure containing about 10% unprocessed BMP-7. Biotinylated soluble BMP-7 complexes were reconstituted by mixing equimolar concentrations of mature BMP-7 that had been biotinylated through the glycan with unmodified prodomain and prodomain that had been biotinylated through amines with unmodified mature domain. Samples were incubated 2 h at RT in 6 M urea, then diluted to 2 M urea in 20 mM Hepes (pH 7.0), 160 mM NaCl, 0.1% Tween-20 both at final protein concentrations of the reconstituted complexes of 2.7 μM. ELISAs with BMP-7 proteins were performed similar to aforementioned AMH, except that Tween-20 was added to all incubation steps at 0.1%. The BMPR2-Fc (catalog no.: 811-BR) and BMP-7 GF mAb (catalog no.: MAB3542) were from R&D Systems.

### Modeling with the equations of Perelson and DeLisi

Data were treated as described in the text and fit to the equations for ligand-induced receptor dimerization (27).

$$\left[\text{R}\right]/\left[{\text{R}}_{\text{0}}\right]=\left(1-\text{β}\right)\left(-1+\text{λ}\right)/2\text{δ}$$

$$\left[\text{RL}\right]/\left[{\text{R}}_{\text{0}}\right]=\text{β}\left(-1+\text{λ}\right)/2\text{δ}$$

$$\left[\text{RLR}\right]/\left[{\text{R}}_{0}\right]=\left(1+2\text{δ}-\text{λ}\right)/4\text{δ}$$

$$\text{β}=\left(\left[\text{L}\right]/K_{1}\right)/\left(1+\left[\text{L}\right]/K_{1}\right)$$

$$\text{δ}=\text{β}\left(1-\text{β}\right)\left[{\text{R}}_{\text{0}}\right]/K_{2}$$

$$\text{λ}={\left(1+4\text{δ}\right)}^{1/2}$$

[L] = ligand concentration

[R_0_] = total concentration of receptor sites

[R] = concentration of free receptor sites at equilibrium

[RL] = concentration of monovalently bound ligand at equilibrium

[RLR] = concentration of bivalently bound ligand at equilibrium

*K*_1_ = dissociation constant for the initial monovalent binding step

*K*_2_ = dissociation constant for the second bivalent binding step

### Modeling of AMH complex dissociation in RL and RLR

To model the equilibria of AMH complex dissociation established during the binding of c-AMH to AMHR2-Fc/Fc, where c-AMH is bound either monovalently (in RL; *K*_*d*_ = *K*_mono_) or bivalently (in RLR; *K*_*d*_ = *K*_bi_), it is necessary to take into account the total amount of prodomain released from both RL and RLR complexes as well as the prodomain released from free c-AMH (*K*_*d*_ = *K*_free_). This is because all three complexes are present in the experiment, and as a common product of dissociation, the total prodomain concentration will determine the position of the equilibria between intact and dissociated complex in all cases. To do this, the modeling was divided into two stages (Fig. S6). In the first stage, the amount of released prodomain (N_free_, N_mono∗_, or N_bi∗_) was calculated at each c-AMH concentration; in the second stage, the level of intact complex, NC_mono_ or NC_bi_, was determined after the association of GF (C_mono_ or C_bi_) with prodomain (N_mono_ or N_bi_) in the presence of prodomain from other sources.

(1) The amount of the prodomain released from c-AMH, RL, or RLR was determined using the quadratic equation for a dissociation/association reaction for the case where the initial concentration of the undissociated complex is known and the complex is allowed to dissociate and come to equilibrium. Specifically, at equilibrium,

$$\left[\text{N}\right]=\left\{-K+{\left(K^{2}+4K\left[{\text{NC}}_{0}\right]\right)}^{1/2}\right\}/2$$

where *K* is the dissociation constant for the complex, [N] is the concentration of the released prodomain, and [NC_0_] is the initial complex concentration.

For the dissociation of free c-AMH, [NC_0_] was set to [c-AMH_0_], and *K* was set to the value of *K*_free_ = 0.4 pM that we determined separately, as described in the main text. For the subsequent analysis, the concentration of prodomain that results from dissociation of free c-AMH was termed [N_free_]. For dissociation of c-AMH bound monovalently to the receptor, as the RL complex, [NC_0_] was set to the value of [RL] calculated using the Perelson and DeLisi equation. As described previously, the concentration of prodomain released (before correction for the presence of prodomain from other sources) was termed [N_mono∗_], and the unknown dissociation constant was termed *K*_mono_. Similarly, for c-AMH bound bivalently in an RLR complex, [NC_0_] was set to the value of [RLR] calculated using the Perelson and DeLisi equation, the concentration of prodomain released (before correction for the presence of prodomain from other sources) was termed [N_bi∗_], and the unknown dissociation constant was termed *K*_bi_.

To calculate [RL] and [RLR] values from [RL]/[R_0_] and [RLR]/[R_0_], it is necessary to specify a concentration for [R_0_]. For this purpose, we used a value of [R_0_] = 0.1 nM, which was determined through experimentation. However, as shown in Fig. S5, values of [R_0_] twice as high (0.2 nM) or half as high (0.05 nM) give similar results when fitting the data to the model, indicating that the results of the analysis described herein are relatively insensitive to errors in the value of [R_0_].

(2) Using the results from (1), the level of association between GF and prodomain, in the presence of prodomain from other sources, was then determined using the quadratic equation for a dissociation/association reaction for the case where the initial concentrations of the two components are known and allowed to associate and come to equilibrium. At equilibrium,

$$\left[\text{NC}\right]=\left\{\left(\left[{\text{N}}_{0}\right]+\left[{\text{C}}_{0}\right]+K\right)-{\left({\left(-\left[{\text{N}}_{0}\right]-\left[{\text{C}}_{0}\right]-K\right)}^{2}-4\left[{\text{N}}_{0}\right]\left[{\text{C}}_{0}\right]\right)}^{1/2}\right\}/2$$

where [N_0_] and [C_0_] represent the initial concentrations of prodomain and GF domain present in the system, *K* is the dissociation constant for the complex, and [NC] is the concentration of intact complex present at equilibrium.

For c-AMH bound monovalently in RL complexes: [C_0_] = [RL] calculated from the Perelson and DeLisi equation as described; [N_0_] = [RL + N_free_ + N_bi∗_]; *K* = *K*_mono_; and [NC], the equilibrium concentration of complex present at equilibrium, is termed [NC_mono_]. For c-AMH bound bivalently in RLR complexes: [C_0_] = [RLR] calculated from the Perelson and DeLisi equation as described; [N_0_] = [RLR + N_free_ + N_mono∗_]; *K* = *K*_bi_; and [NC] is termed [NC_bi_].

The levels of C-terminal GF without a prodomain, in the RL and RLR complexes, and the N-terminal prodomain released from the RL and RLR complexes were then derived as followed:

$$\left[{\text{C}}_{\text{mono}}\right]=\left[{\text{N}}_{\text{mono}}\right]=\left[\text{RL}\right]-\left[{\text{NC}}_{\text{mono}}\right]\text{ and }\left[{\text{C}}_{\text{bi}}\right]=\left[{\text{N}}_{\text{bi}}\right]=\left[\text{RLR}\right]-\left[{\text{NC}}_{\text{bi}}\right]$$

And the fraction intact complex, F, is given by:

$$\text{F}=\left[{\text{NC}}_{\text{mono}}+{\text{NC}}_{\text{bi}}\right]/\left[\text{RL}+\text{RLR}\right]=\left[{\text{NC}}_{\text{T}}\right]/\left[\text{RL}+\text{RLR}\right]$$

(3) The quantities defined in (1) and (2) were then used to define a notional composite equilibrium constant for the combined dissociation of RL and RLR, which we termed *K*_T_.

$$K_{\text{T}}=\left[{\text{N}}_{\text{mono}}+{\text{N}}_{\text{bi}}\right]\left[{\text{C}}_{\text{mono}}+{\text{C}}_{\text{bi}}\right]/\left[{\text{NC}}_{\text{mono}}+{\text{NC}}_{\text{bi}}\right]$$

$$K_{\text{T}}=\left[{\text{N}}_{\text{T}}\right]\left[{\text{C}}_{\text{T}}\right]\text{/}\left[{\text{NC}}_{\text{T}}\right]$$

*K*_T_ is not a real equilibrium constant but instead reflects the overall distribution of intact *versus* dissociated complex for a given mixture of RL and RLR. However, because *K*_T_ relates directly to measured experimental quantities, we could fit the measured values of *K*_T_ at each total AMH concentration against the aforementioned equation to determine the best-fit values for *K*_mono_ and *K*_bi_.

### Calculation of P_a_

The fraction of AMH in the RL and RLR complexes bound by mAb-C_1_ (P_a_) was calculated using the quadratic equation for a reversible association reaction:

$${\text{P}}_{\text{a}}=\left[{\text{mAb}\text{-}\text{C}}_{1}\text{-}\text{AMH}\;\text{complex}\right]/\left[\text{RL}+\text{RLR}\right]=\left[-\text{b}-{\left({\text{b}}^{2}-4\text{c}\right)}^{1/2}\right]/2\left[\text{RL}+\text{RLR}\right]$$

where b = −[RL + RLR] − [mAb-C_1_] − *K*_*d*_ and c = [RL + RLR][mAb-C_1_].

The *K*_*d*_ for the AMH–mAb-C_1_ interaction is 0.1 nM.

## Data availabilty

All data described in the article are contained within the article.

## Supporting information

This article contains supporting information.

## Conflict of interest

The authors declare that they have no conflicts of interest with the contents of this article.
