# Supplementary material for: The anti-Müllerian hormone prodomain is displaced from the hormone/prodomain complex upon bivalent binding to the hormone receptor
Source: J Biol Chem. 2021 Nov 19;298(1):101429. doi: 10.1016/j.jbc.2021.101429 (PMC8801479; doi:10.1016/j.jbc.2021.101429)
Supplement: Supplemental Figures S1–S6 [file mmc1.docx]

**SUPPLEMENTARY INFORMATION**

**Title:** The anti-Müllerian hormone prodomain is displaced from the hormone/prodomain complex upon bivalent binding to the hormone receptor

**Authors**: Richard L. Cate, Nathalie di Clemente, Chrystèle Racine, Nigel P. Groome, R. Blake Pepinsky, and Adrian Whitty

**Supplementary Figure Legends**

**Supplementary Figure S1. Reducing the level of mAb-C_1_ bound by RLR and RL eliminates the 2/1 detection bias observed when mAb-C_1_ and the secondary Ab are added individually in separate steps.** At high concentrations of mAb-C_1_, RL and RLR complexes should each have two molecules of mAb-C_1_ bound. Our hypothesis is that both mAbs bound to RLR are detectable by the secondary Ab, but only one mAb bound to RL is detectable, generating a 2/1 detection bias (depicted in Supplementary Figure S2C). If correct, decreasing mAb concentration or diluting mAb with Fab should reduce and ultimately eliminate this 2/1 detection bias, because the fraction of RL and RLR complexes with two molecules of mAb-C_1_ bound will be reduced eventually to zero. **(A,D)** Diagrams depict strategies for lowering the level of mAb-C_1_ bound by RL and RLR, either by diluting the mAb-C_1_ concentration to or below the K_d_ for the AMH/mAb-C_1_ interaction (K_d_ ≅ 0.1 nM) **(A),** or by diluting mAb-C_1_ with Fab **(D).** The equations for modeling the effects of decreasing mAb concentration or diluting mAb with Fab are shown. The fraction of RL and RLR complexes with one or two mAbs bound at lower [mAb-C_1_] or after dilution with Fab can be predicted using the Hardy Weinberg equation for genetic variation at a locus with two alleles. When [mAb-C_1_] is decreased, the fractions of AMH bound by mAb-C_1_ or free can be used in place of the two allele frequencies, and an expression derived for how a decrease in mAb-C_1_ concentration affects the detection of mAb-C_1_ bound to RL and RLR, shown as “B” in **A.** The fraction of AMH bound by mAb-C_1_ (P_a_) can be calculated with the quadratic equation for a simple association reaction using the specified concentration of mAb-C_1_, the concentration of AMH bound to AMHR2, [RL+RLR], and the K_d_ for the AMH/mAb-C_1_ interaction (see Experimental Procedures). When mAb-C_1_ is diluted with Fab, the fractions of mAb and Fab can be used in place of the two allele frequencies, and a similar expression derived for how dilution with Fab affects the detection of mAb-C_1_ bound to RL and RLR, shown as “B” in **D**. In this situation, an additional term is required (Z) because the effect of diluting mAb-C_1_ with Fab is less for AMH bound monovalently (RL) than bivalently (RLR). “Z” is the ratio of the dilution factors for mAb-C_1_ bound to RL and RLR. **(B,E)** Graphs showing the affects of decreasing mAb-C_1_ concentration **(B)** or of diluting mAb-C_1_ with Fab **(E)**, using the equations in **A** or **D**, respectively. **(C)** Results of experiments in which the concentration of mAb-C_1_ was reduced to close to the K_d_ for the AMH/mAb-C_1_ interaction. The data for mAb-C_1_ at the high concentration fit to [RL+2RLR]/[R_0_], while data at the low concentration fit to [RL+RLR]/[R_0_]. A significant difference was not observed when mAb-N_1_ was tested at the high and low concentrations. **(F)** Results of experiments in which mAb-C_1_ was diluted with Fab by a factor of 4. The undiluted mAb-C_1_ data fit to [RL+2RLR]/[R_0_], while the data with mAb-C_1_ diluted with Fab fit to [RL+0.65RLR]/[R_0_]. Only a minimal affect was observed when mAb-N_1_ is diluted with Fab. K_1_ and [R_0_]/K_2_ values were determined by simultaneously fitting all data to the predicted curves and minimizing the RMSE. Error bars show standard deviations. **Conclusion**: These results are consistent with the secondary Ab being able to detect two molecules of mAb-C_1_ bound to RLR, but only one of the two molecules bound to RL. They are not consistent with an alternative hypothesis that two molecules of mAb-C_1_ can bind to RLR, but only one can bind to RL. If that were the case, decreasing the [mAb-C_1_] or dilution of mAb with Fab would not have an effect on the 2/1 detection bias.

**Supplementary Figure S2. RLR and RL only bind one molecule of the mAb-C_1_ – secondary Ab complex.** Raw absorbance data for an ELISA plotted on a logarithmic **(A)** and linear scale **(B)**. The reason that the RLR/RL 2 to 1 bias is eliminated when mAb-C_1_ and the secondary Ab are presented as a complex could be due to either both RLR and RL being able to bind two molecules of the complex or only one molecule of the complex. If both RLR and RL bind 2 molecules of the complex, then at a high concentration of AMH where almost all of the AMH is bound monovalently, the absorbance should be twice as high as when mAb-C_1_ and the secondary Ab are added individually. If both RLR and RL bind only one molecule of the complex, the absorbances should be equal. As shown in A and B, the absorbances at high AMH concentration are almost equal with the two conditions, indicating that both RLR and RL only bind one molecule of the complex. **(C)** The different stoichiometries of the secondary Ab (mediated by mAb-C_1_) binding to RLR and RL are shown for when mAb-C_1_ and the secondary Ab are added as a complex (1 to 1) and individually (2 to 1). K_1_ and [R_0_]/K_2_ were determined as in Figure 5. Error bars show standard deviations.

**Supplementary Figure S3.** **Relationships between the signals detected by mAb-N_1_ and mAb-C_1_ and the concentrations of RL, RLR, and other species.** **(A)** The diagram shows the two equilibrium states for the AMH complex interacting with AMHR2 monovalently (RL) or bivalently (RLR). Formulas for calculating concentrations of species and additional parameters are shown below the diagram, and are similar to the calculations shown in Table 1 except that in the table, ratios of concentrations of species relative to [R_0_] are shown. The color scheme used for the equations is identical to the one used in Figures 5A and 5C. **(B**) Effect of using [bound mAb-N_1_] as an estimate of [RL] and for calculating levels of [RLR]. [bound mAb-N_1_] = [NC_T_], but when K_mono_ is small and K_bi_ is large, [bound mAb-N_1_] ≈ [RL]. Solid lines were generated with the equations of Perelson and DeLisi; dashed lines were generated using the equations of Perelson and DeLisi in combination with the quadratic equation for a reversible reaction. [NC_T_] was used in place of [RL] for the RL/R_0_ estimate and [RL+RLR] - [NC_T_] was used in place of [RLR] for the RLR/R_0_ estimate. The values of K_1_, [R_0_]/K_2_, K_mono_, and K_bi_ used for the simulation are shown.

**Supplementary Figure S4. Additional results supporting the relationship between [bound mAb-C_1_] and [RL + RLR] or [RL + 2RLR] depending on the experimental conditions and between [bound mAb-N_1_] and [RL].** **(A)** The results with mAb-N_2_ and mAb-C_2_ are plotted with the results of mAb-N_1_ and mAb-C_1_ shown in Figure 5C and Table 1 to allow a direct comparison. mAb-N_2_ and mAb-C_2_ bind different epitopes than mAb-N_1_ and mAb-C_1_ in the prodomain and GF domains, respectively, but give similar results. **(B)** The results with biotinylated c-AMH are plotted with the results of mAb-N_1_ shown in Figure 5C and Table 1 to allow a direct comparison. Biotinylated-c-AMH, in which most of the biotin label is in the prodomain, gives similar results as mAb-N_1_, which detects the prodomain in RL. ELISA formats are shown below each graph. K_1_ and [R_0_]/K_2_ values were determined by simultaneously fitting all data to the predicted curves and minimizing the RMSE. Error bars show standard deviations.

**Supplementary Figure S5. K_mono_ and [R_0_]/K_2_ values derived from fitting experimental K_T_ values to those obtained via modeling are in close agreement over a range of [R_0_] values.** The modeling was performed for AMH complex dissociation as described in the Experimental Procedures. The data are from Table 1 (column F), adjusted for the appropriate value of [R_0_]. The graph in **B** ([R_0_] = 0.1 nM) is identical to the graph shown in Figure 7C to allow a direct comparison with the graphs in **A** ([R_0_] = 0.2 nM) and **C** ([R_0_] = 0.05 nM). In the table below the graphs, K_1_ and K_bi_ were fixed at 1.1 nM and 5.0 nM, respectively, along with the indicated value of [R_0_], and values for K_mono_ and K_2_ were determined by minimizing the RMSE. Experiments have indicated that the level of AMHR2 captured in the ELISA plate well (i.e [R_0_]) is around 0.1 nM. However, K_mono_ and [R_0_]/K_2_ values obtained at [R_0_] = 0.2 nM and [R_0_] = 0.05 nM are in fairly close agreement with those obtained at [R_0_] = 0.1 nM, indicating that an exact value of [R_0_] is not essential for this analysis.

**Supplementary Figure S6. Modeling of AMH complex dissociation in RL and RLR.** The two stages of modeling are described in the Experimental Procedures. The equations for calculating the level of released prodomain, [N], in the first stage, and the level of intact complex, [NC], in the second stage, are shown. Substituting values of [N_mono_] and [N_bi_] obtained in Stage 2 for the values of [N_mono*_] and [N_bi*_] obtained in Stage 1 has a negligible effect on [NC_mono_] and [NC_bi_] values and therefore little effect on the calculation of K_T_. For all modeling exercises, the K_d_ for c-AMH in solution, K_free_, was fixed at 0.4 pM.

**Supplementary Figure S1**

**
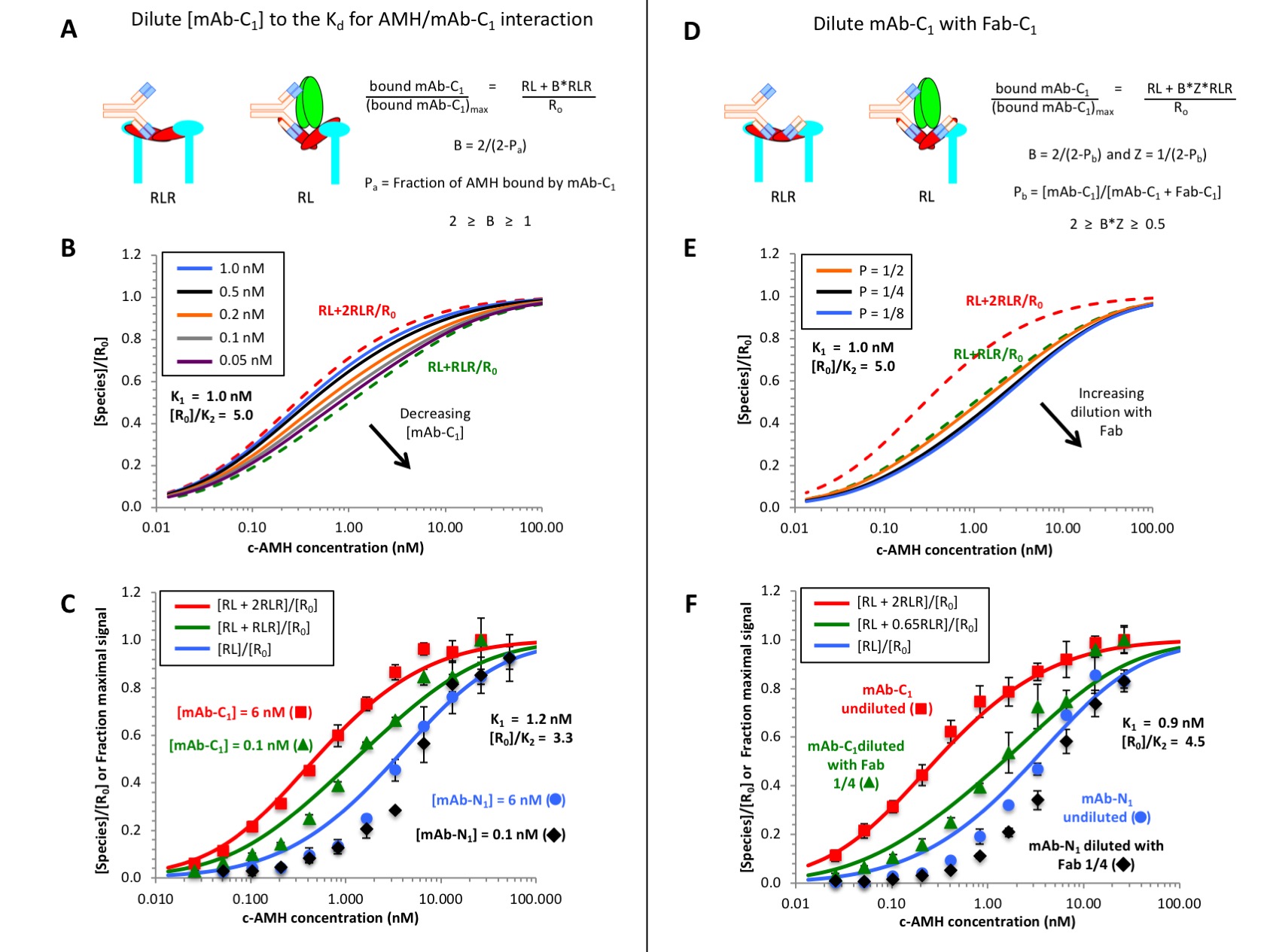
**

**Supplementary Figure S2**

**
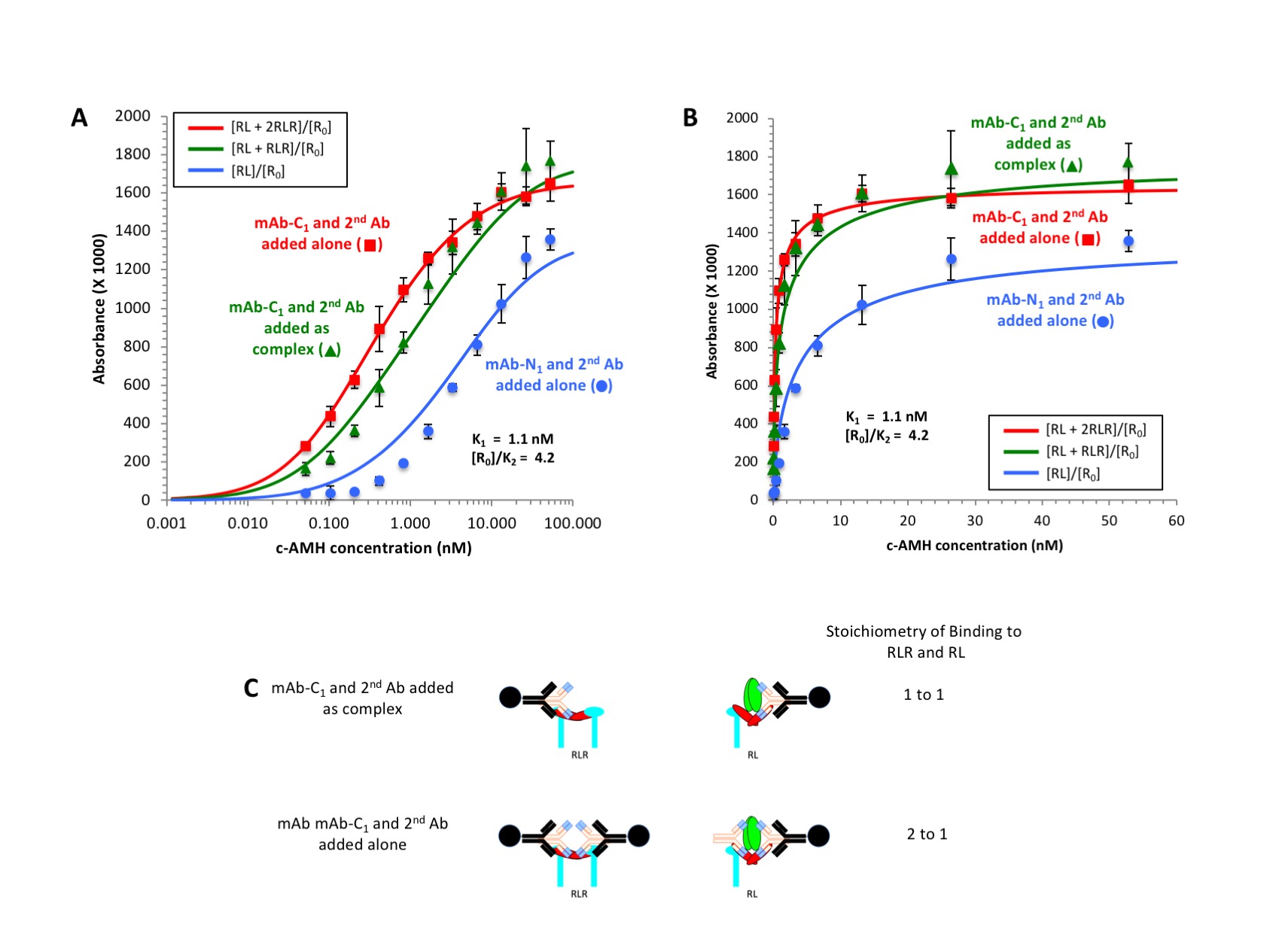
**

**Supplementary Figure S3**

**
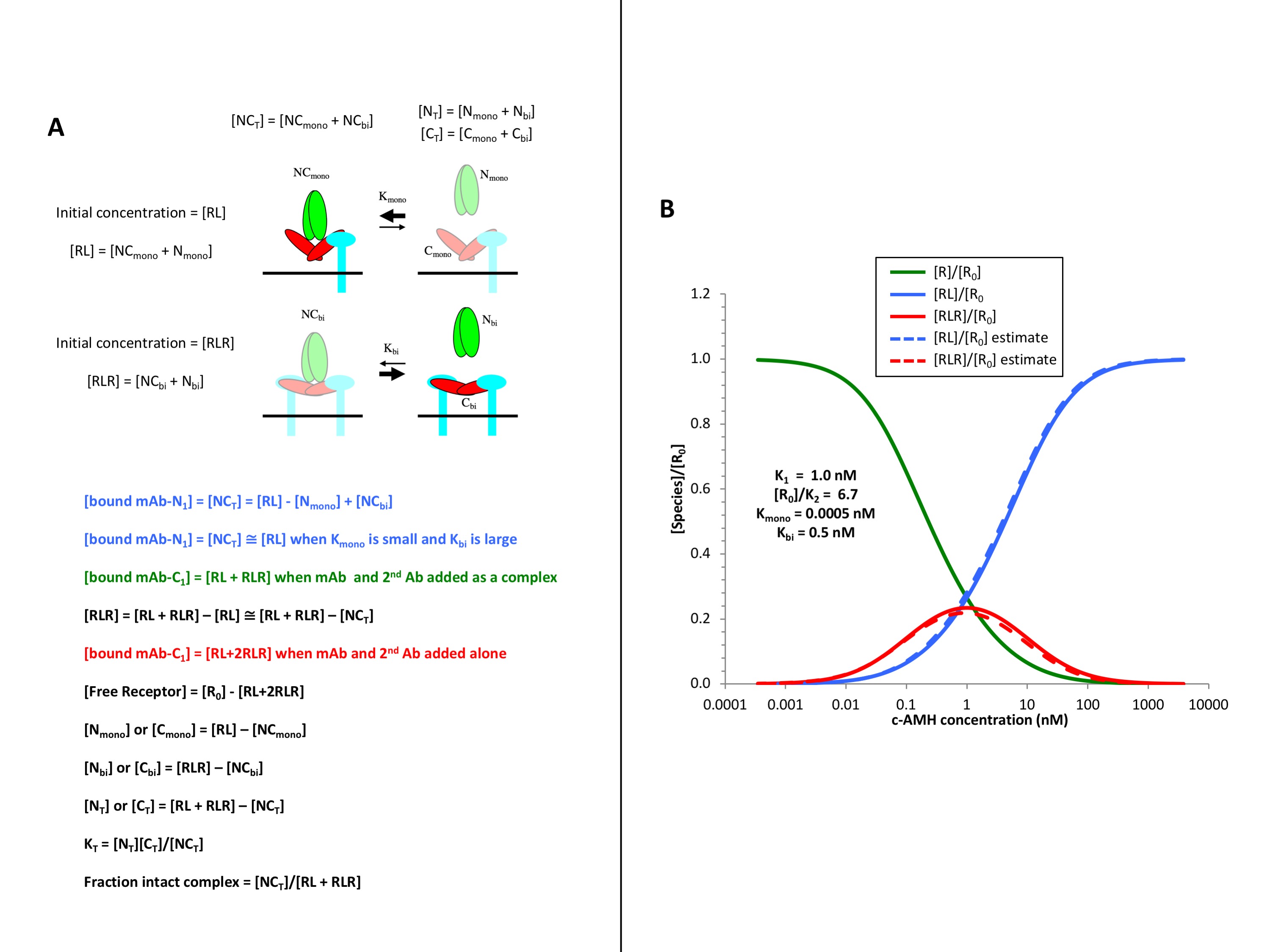
**

**Supplementary Figure S4**

**
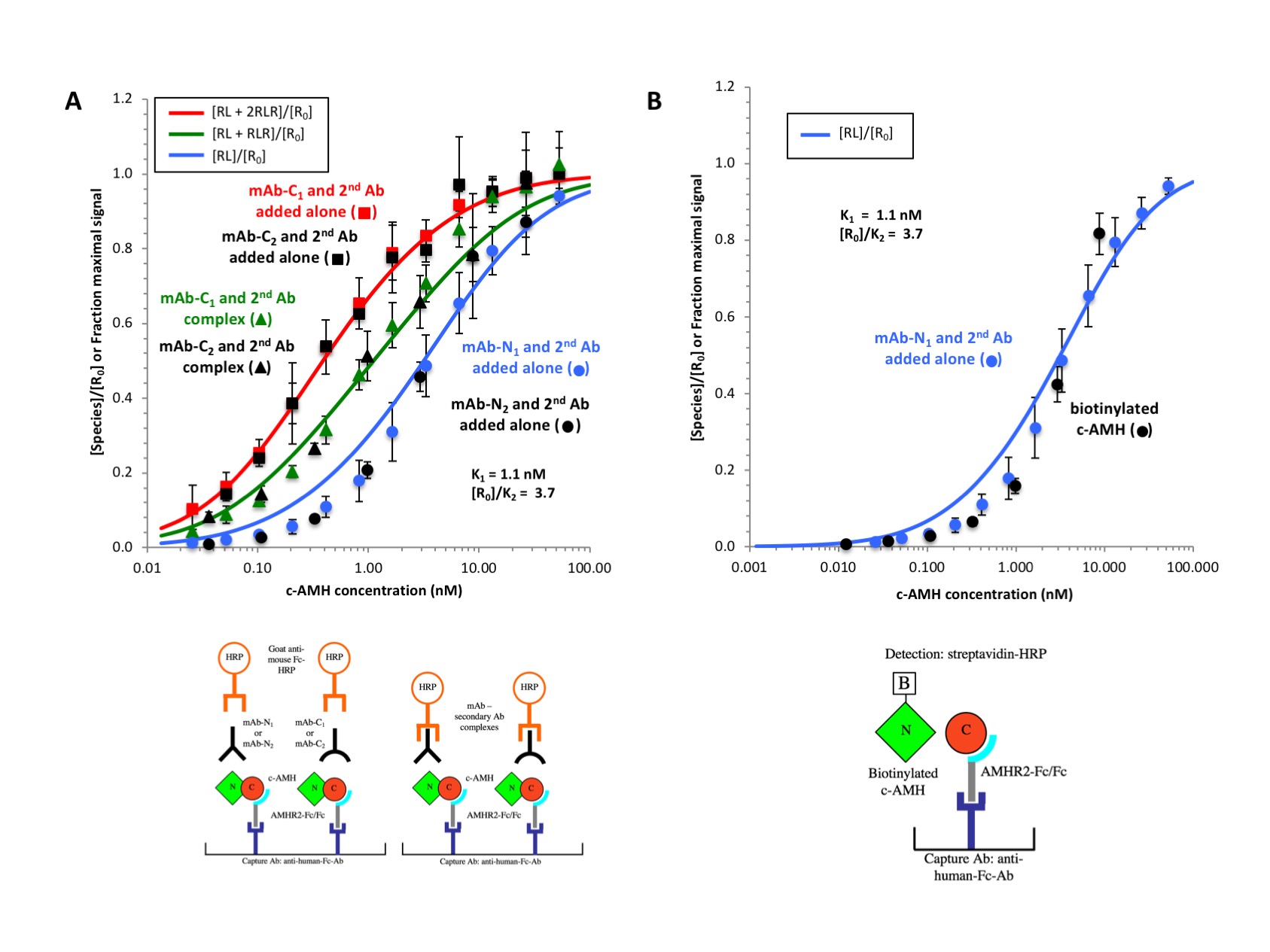
**

**Supplementary Figure S5**

**
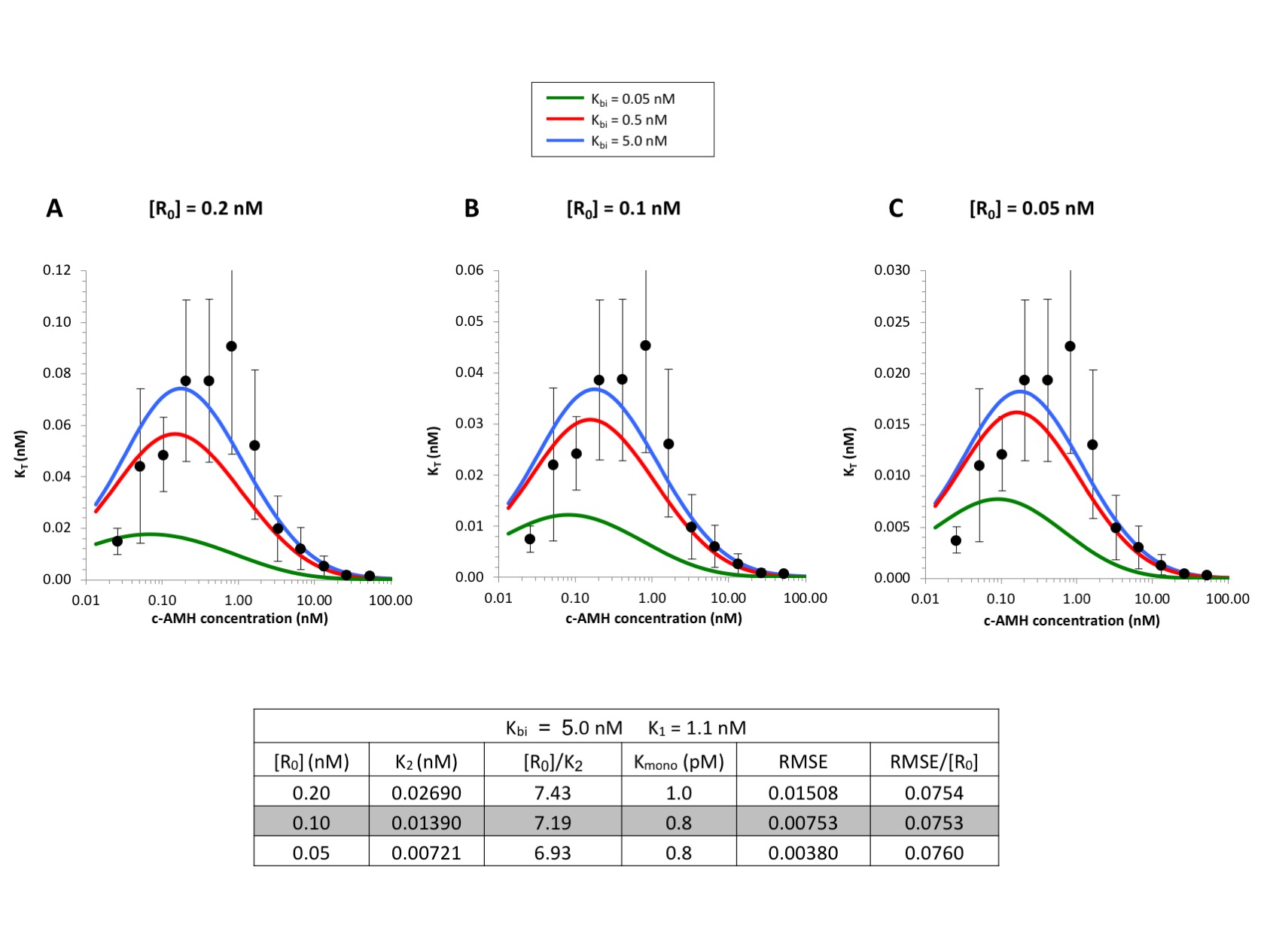
**

**Supplementary Figure S6**

**
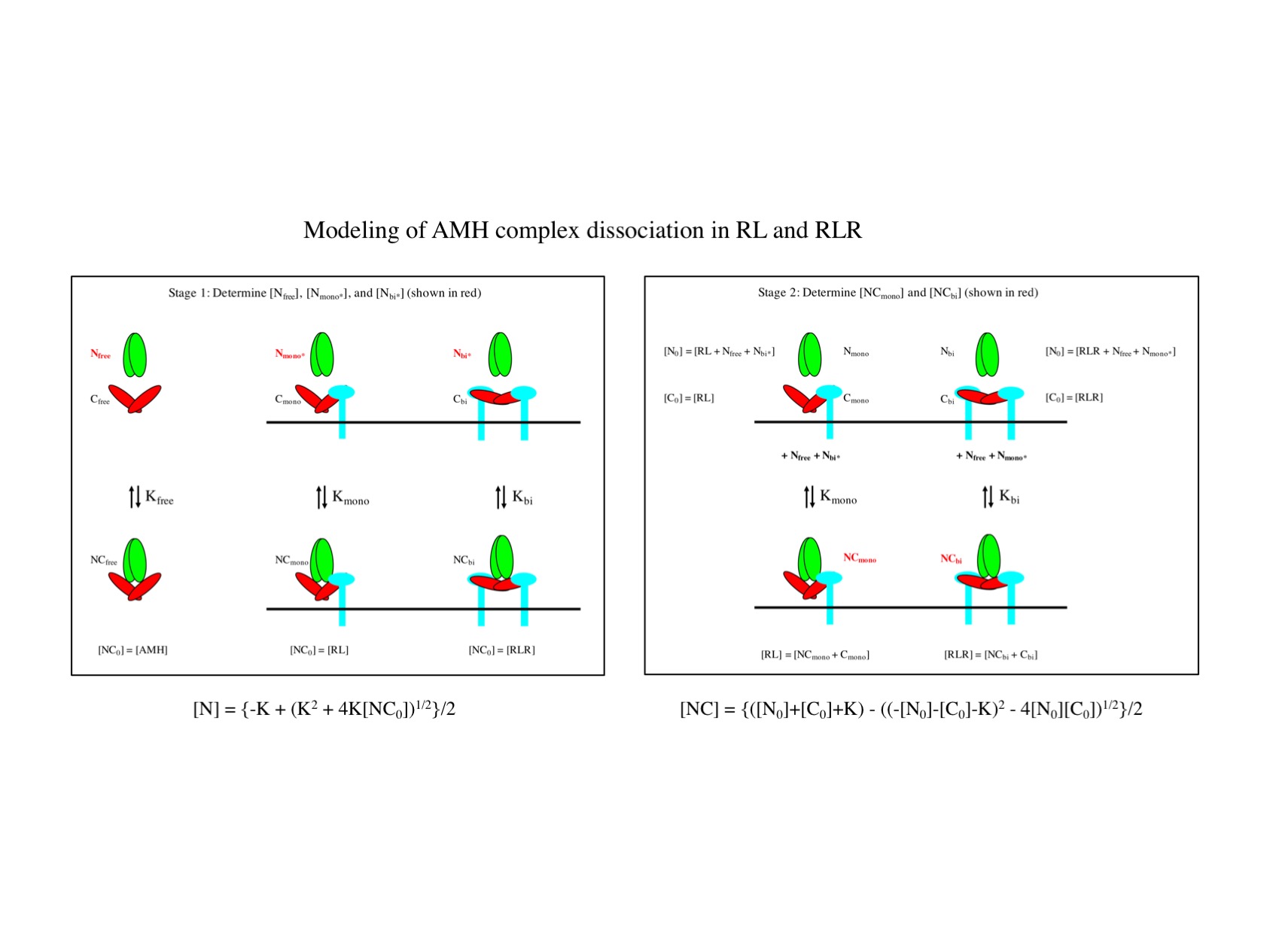
**
